# Design, Synthesis, and Biological Evaluation of Novel Triazine‐Based Dual Histone Deacetylase/phosphatidylinositol 3‐kinase Inhibitors for Breast Cancer Therapy

**DOI:** 10.1002/cmdc.202501041

**Published:** 2026-04-25

**Authors:** Lara Luzietti, Gustavo Salgado Pires, Ana Ryan, Crisamie Regidor, Matthew Hiller, Danilo Sarti, Tríona Ní Chonghaile, Pedro de Sena Murteira Pinheiro, Damir Varešlija, Daniel Alencar Rodrigues

**Affiliations:** ^1^ School of Pharmacy and Biomolecular Sciences (PBS) Royal College of Surgeons in Ireland Dublin Ireland; ^2^ Laboratório de Avaliação e Síntese de Substâncias Bioativas (LASSBio) Instituto de Ciências Biomédicas Universidade Federal do Rio de Janeiro Cidade Universitária Rio de Janeiro Brazil; ^3^ Programa de Pós‐Graduação em Farmacologia e Química Medicinal (PPGFQM) Instituto de Ciências Biomédicas Universidade Federal do Rio de Janeiro Cidade Universitária Rio de Janeiro Brazil; ^4^ Department of Physiology and Medical Physics Royal College of Surgeons in Ireland Dublin Ireland

**Keywords:** breast cancer, cancer, histone deacetylase (HDAC), phosphatidylinositol 3‐kinase (PI3K), triazine

## Abstract

Breast cancer is the most frequently diagnosed malignancy and a leading cause of cancer‐related mortality among women worldwide. Triple‐negative breast cancer (TNBC) poses a major clinical challenge due to its aggressive nature, limited therapeutic options, and high propensity for drug resistance. Dysregulation of the phosphatidylinositol 3‐kinase (PI3K)/AKT/mTOR and histone deacetylase (HDAC) signaling pathways has been implicated in TNBC progression and therapeutic resistance, highlighting their potential as combinatorial targets. In this study, we report the design, synthesis, and biological evaluation of a novel series of triazine‐based multitarget inhibitors aimed at the dual inhibition of PI3K and HDAC. Among the synthesized compounds, **5b** and **5f** demonstrated the most promising profiles, exhibiting low nanomolar IC_50_ values against HDAC6 (2.33 and 6.02 nM) and PI3Kα (17.5 and 236 nM), respectively. Both compounds reduced cell viability in breast cancer cell lines, with IC_50_ values below 5 µM in MDA‐MB‐231 cells. Western blot analysis confirmed inhibition of HDAC and PI3K signaling in treated cells. Molecular docking and dynamics simulations further revealed stable binding modes and favorable interactions within the active sites of both targets. Overall, **5b** and **5f** represent promising lead candidates for further optimization toward the development of novel dual HDAC/PI3K inhibitors with potential application in TNBC therapy, as evaluated in TNBC‐relevant models.

## Graphical abstract

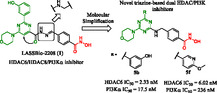

## 1 Introduction

Breast cancer is the most commonly diagnosed malignancy and a leading cause of cancer‐related mortality among female patients worldwide. In 2022 alone, ≈ 2.3 million women were diagnosed with the disease, resulting in an estimated 670,000 deaths globally [1]. Despite substantial advances in early detection, surgical interventions, targeted therapy, and chemotherapy, survival rates for patients with recurrent or metastatic disease have not significantly improved, underscoring the urgent need for more effective, durable, and targeted therapeutic strategies [1, 2]. Among breast cancer subtypes, TNBC is one of the most complex clinical challenges. It represents approximately 10%–15% of all diagnosed breast cancers and is defined by the lack of estrogen receptor (ER), progesterone receptor (PR), and human epidermal growth factor receptor 2 (HER2) expression, rendering it unresponsive to both hormone‐based and HER2‐targeted therapies [3]. Clinically, TNBC is highly aggressive, characterized by early metastasis, high recurrence rates, and poor overall prognosis, with the 5‐year survival rates for metastatic cases remaining below 30% [3]. The therapeutic landscape is further complicated by the tumor's pronounced heterogeneity and its propensity to rapidly develop resistance to conventional chemotherapeutic agents, which remain the cornerstone of treatment [3].

Molecular profiling has revealed that TNBC frequently exhibits aberrations in several signaling cascades, including the phosphatidylinositol 3‐kinase (PI3K)/AKT/mTOR, MAPK/ERK, and JAK/STAT pathways [4]. Among these, the PI3K/AKT/mTOR pathway, which plays a central role in regulating cell survival, metabolism, and proliferation, is altered in up to 30% of all TNBC cases [3, 4, 5]. Combination therapies involving AKT inhibitors and chemotherapeutic agents such as paclitaxel have demonstrated clinical benefit in tumors with PI3KCA, AKT, or phosphatase and tensin homologue (PTEN) alterations, offering a promising therapeutic avenue for subsets of TNBC patients [3]. These findings underscore the therapeutic relevance of targeting the PI3K pathway. Although several PI3K inhibitors have gained regulatory approval (e.g., idelalisib, copanlisib, duvelisib, alpelisib, and umbralisib), their clinical use has been limited by toxicity, off‐target effects, and the emergence of resistance mechanisms [6, 7]. As a result, while the pathway remains a viable target, further optimization is necessary to enhance efficacy and tolerability.

**FIGURE 1:**
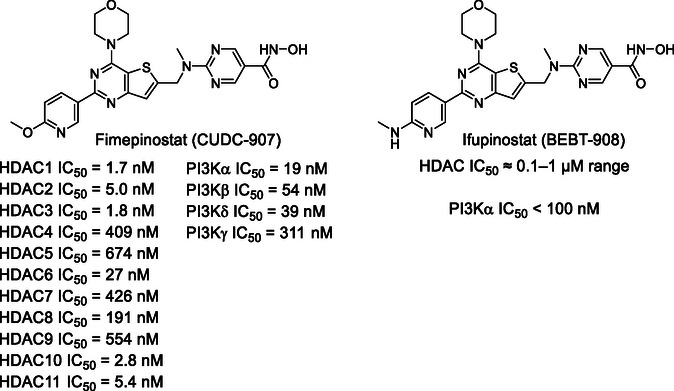
Chemical structures of fimepinostat [17] and ifupinostat [20].

One mechanism underlying resistance to PI3K‐targeted therapies is compensatory activation of parallel oncogenic pathways, including histone deacetylase (HDAC)‐mediated signaling [8, 9, 10]. Histone deacetylase 6 (HDAC6), a cytoplasmic deacetylase, regulates protein homeostasis, cell motility, immune evasion, and stress response by modulating substrates such as α‐tubulin and HSP90 [11, 12, 13]. Importantly, HDAC6 inhibition has shown the potential to impair tumor cell migration, enhance immune surveillance, and sensitize tumors to other treatments [11, 12, 14, 15]. Emerging evidence suggests that simultaneous inhibition of PI3K and HDAC pathways can yield synergistic antitumor effects by disrupting compensatory feedback mechanisms and overcoming acquired resistance [8, 10, 16]. This rationale has driven the development of multitarget inhibitors that incorporate both activities into a single molecular entity, with encouraging results in both preclinical and clinical settings. Notably, compounds such as fimepinostat (CUDC‐907) [17, 18] and ifupinostat (BEBT‐908) [19] (Figure 1) have progressed to clinical trials, where they have demonstrated favorable safety profiles and promising therapeutic efficacy. CUDC‐907, a dual PI3K and pan‐HDAC inhibitor, has shown greater efficacy across tumor models compared to either monotherapy or conventional combination regimens [17, 18]. However, dose‐limiting toxicities and off‐target effects associated with pan‐HDAC inhibition have limited its broader clinical utility [18, 21]. Recently, ifupinostat has been approved in China for treatment of relapsed or refractory diffuse large B‐cell lymphoma [22], highlighting the promising profile of the multitarget inhibition of PI3K and HDACs.

These findings underscore the therapeutic potential of more selective multitarget approaches. In particular, dual inhibition of PI3K and HDAC6 represents a refined strategy aimed at maximizing anticancer efficacy while reducing toxicity [23, 24]. By designing single‐molecule inhibitors with selective activity against PI3K and HDAC6, it may be possible to overcome limitations of current therapies and achieve more durable responses in TNBC and other cancers with co‐dysregulation of these pathways. Herein, we describe the design, synthesis, and biological evaluation of novel triazine‐based dual HDAC/PI3K inhibitors for breast cancer therapy.

## 2 Results and Discussion

### 2.1 Design

**FIGURE 2:**
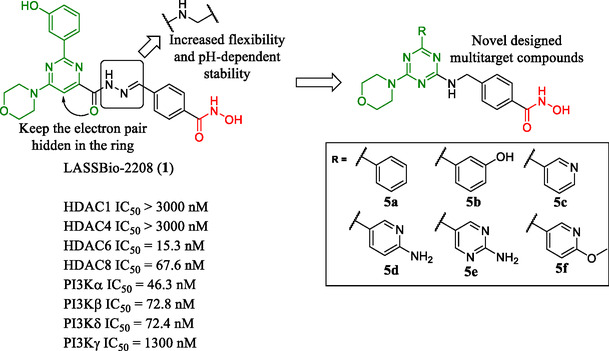
Design concept of novel multitarget inhibitors of HDAC6 and PI3K. Green subunit represents the pharmacophore for PI3K inhibition, and in red, the hydroxamic acid is key for interaction with HDACs.

Our previous studies focused on the design of multitarget HDAC6/PI3K inhibitors based on the structure of LASSBio‐2208 (**1**), an *N*‐acylhydrazone (NAH) derivative shown to act as a dual HDAC6/8 and PI3Kα inhibitor [25]. However, the NAH motif in this compound was found to be acid‐labile, limiting its stability. To address this, we applied a molecular simplification strategy [26] by hiding the electron pair of the oxygen atom in the pyrimidine ring, thereby exploring a 1,3,5‐triazine system. Furthermore, we replaced the hydrazone moiety with a methyleneamine, which increases the flexibility and pH‐dependent stability of the final compounds (Figure 2). To interact with PI3K, we explored the use of morpholine as a hinge‐binding motif [27]. To enable interactions with the affinity pocket of PI3Ks, we designed a focused library by incorporating aromatic and heteroaromatic rings to explore potential interactions with Tyr836, Asp810, and Lys802 residues [28, 29]. We utilized the phenyl ring (**5a**) as a negative control for PI3K inhibition, whereas the focused set comprising 3‐hydroxyphenyl (**5b**), pyridin‐3‐yl (**5c**), 6‐aminopyridin‐3‐yl (**5d**), 2‐aminopyrimidin‐5‐yl (**5e**), and 6‐methoxypyridin‐3‐yl (**5f**) was selected based on prior structure–activity relationship studies of PI3K inhibitors [17, 18, 23, 30, 31]. Finally, we employed a hydroxamic acid moiety as the zinc‐binding group (ZBG) for HDAC inhibition and introduced a phenyl linker to promote selective inhibition of HDAC6 [13, 32, 33].

### 2.2 Chemistry

**SCHEME 1:**
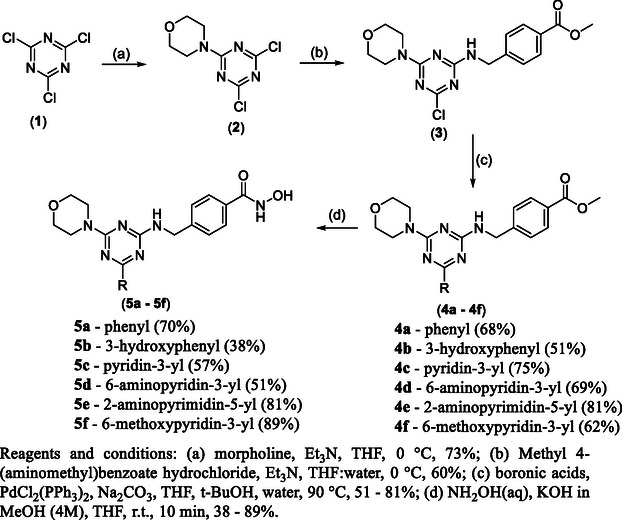
Synthesis of triazine‐based HDAC and PI3K multitarget inhibitors.

The synthesis of the multitarget inhibitors is shown in Scheme 1; we started with cyanuric chloride (**1**), which underwent consecutive nucleophilic aromatic substitutions with morpholine and methyl 4‐(aminomethyl)benzoate hydrochloride, affording 4‐(4,6‐dichloro‐1,3,5‐triazin‐2‐yl)morpholine (**2**) and methyl 4‐(((4‐chloro‐6‐morpholino‐1,3,5‐triazin‐2‐yl)amino)methyl)benzoate (**3**) in 73% and 60% yield, respectively. After that, the intermediate (**3**) was submitted to a Suzuki coupling reaction to afford **4a‐4f** in 51–81% yield. Finally, the reaction of **4a‐4f** with an aqueous solution of hydroxylamine under basic conditions [34] afforded the hydroxamic acids **5a‐5f** in 38‐89% yield.

**FIGURE 3:**
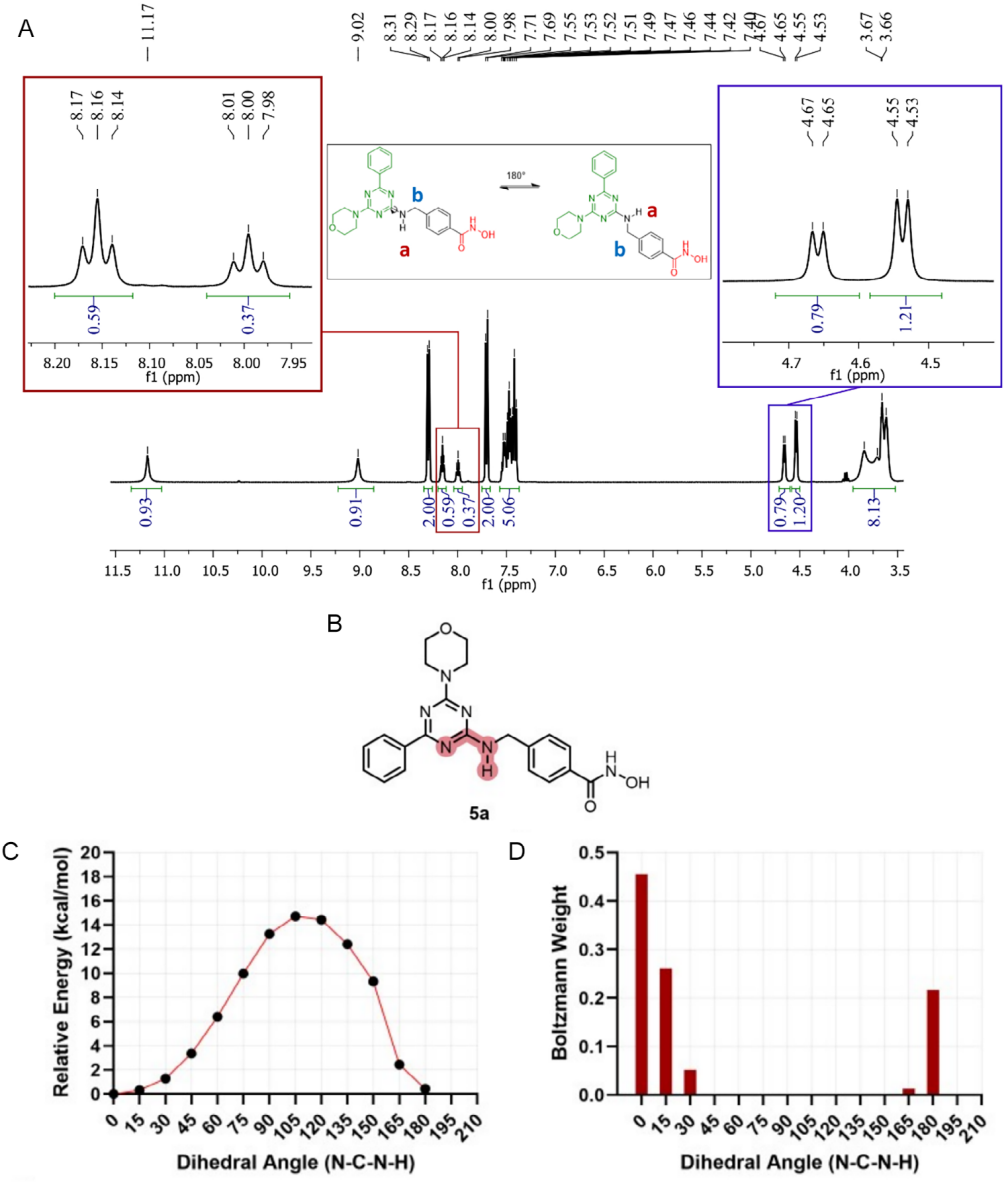
(A) Hydrogen nuclear magnetic resonance of compound **5a** (400 MHz, DMSO‐d_6_) showing the presence of two conformers in solution. (B) Structure of derivative **5a** used in the analysis, with the evaluated dihedral angle highlighted. (C) Conformational analysis of derivative **5a** through dihedral angle scanning and energetic evaluation. Potential energy surface (PES) scan of the N–C–N–H dihedral angle (0°–180°, 15° steps) calculated at the ωB97XD/6‐31+G(d,p) level, C‐PCM solvation model for polar organic solvents (ε = 37). (D) Boltzmann distributions of the identified conformers as a function of their relative energies at room temperature.

Hydrogen nuclear magnetic resonance analysis of this series revealed duplication in some signals, which could be due to tautomerism (Table S1), or the compounds could exist as two conformations in solution, likely due to rotation around the guanidine‐like bond (Figure 3A). For further investigation, we used compound **5a** as example. The tautomeric stability analysis of compound **5a**, performed through full geometry optimization at the ωB97XD/6‐31+G(d,p) level of theory, revealed the predominance of a single tautomeric form among the eight plausible tautomers generated, corresponding to the representation employed throughout this study (Table S1). Furthermore, the relaxed potential energy surface (PES) scan conducted for **5a** indicated the existence of two distinct rotameric populations, defined by the dihedral angle N–C–N–H (Figure 3B), and separated by an energy barrier of 14.72 kcal·mol^−1^, as illustrated in Figure 3C. A Boltzmann distribution ratio of 7:3 was observed between the conformers with dihedral angles of 0° and 180°, respectively (Figure 3D). This finding is consistent with the duplicated NMR signals experimentally observed (6:4) (Figure 3A), since thermal energy input is required to enable interconversion between these two populations, thereby generating distinct chemical environments.

### 2.3 Biological Evaluation

**FIGURE 4:**
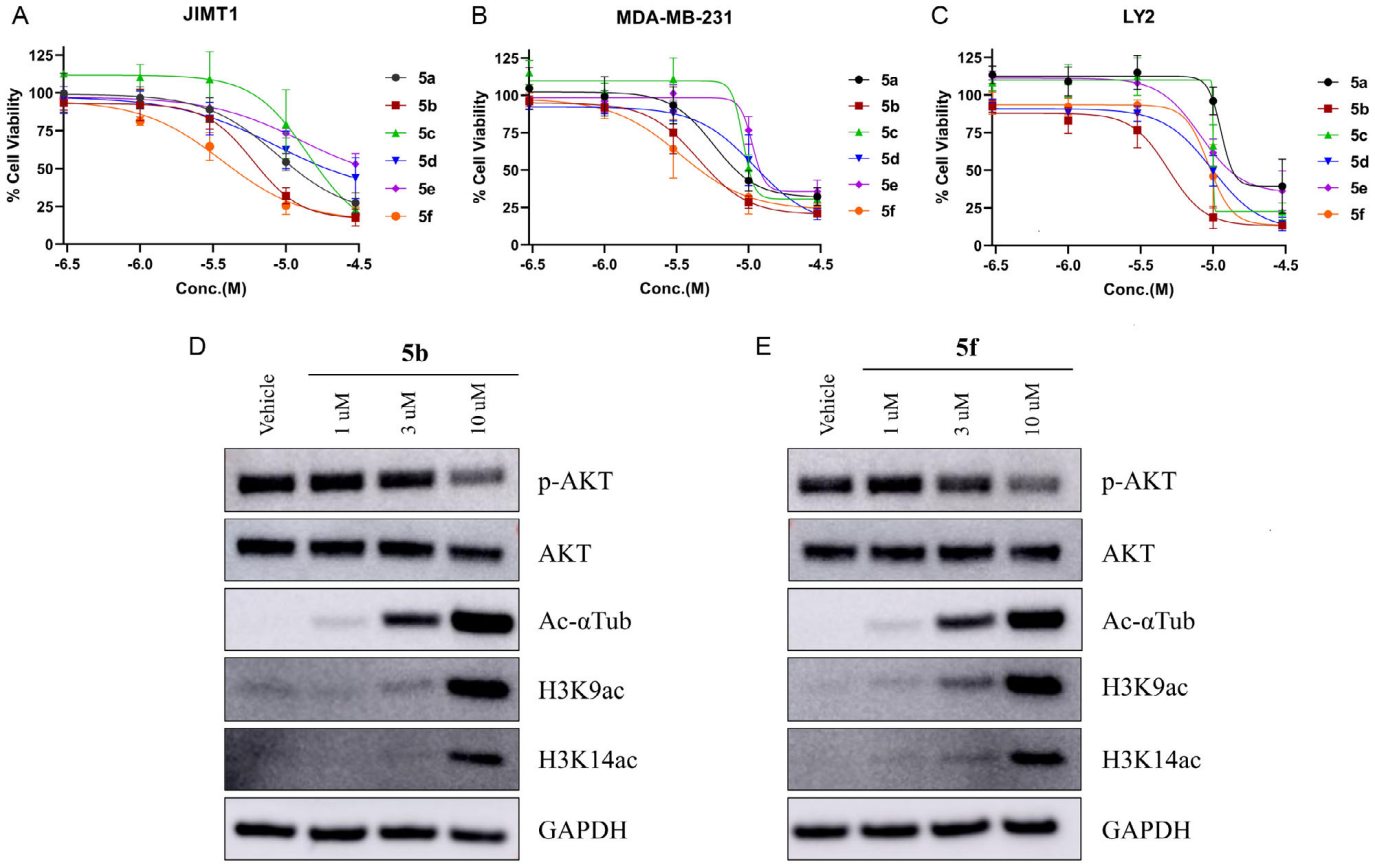
Cell‐based evaluation of compounds **5a–** **5f**. Dose‐dependent effects of compound treatment (**5a**‐**5f**) on breast cancer cell viability across JIMT1 (A), MDA‐MB‐231 (B), and LY2 (C). Cell‐based evaluation of HDAC and PI3K inhibition by compounds **5b** and **5f** by Western blot. JIMT1 cells were treated for 24 h with compounds **5b** (D) and **5f** (E) at the concentrations of 1, 3, and 10 µM or with vehicle control (0.1% DMSO). Protein expression levels were analyzed by western blot using antibodies against acetylated α‐tubulin, acetyl H3 (H3K9Ac and H3K14Ac), phospho‐AKT (p‐AKT), and total AKT. GAPDH served as a loading control.

The cell viability effects of compounds **5a–5f** were assessed in LY2, JIMT1, and MDA‐MB‐231 breast cancer cell lines using the 3‐(4,5‐dimethylthiazol‐2‐yl)‐5‐(3‐carboxymethoxyphenyl)‐2‐(4‐sulfophenyl)‐2*H*‐tetrazolium (MTS) assay**.** The MTS assay measures metabolic viability and serves as a first‐pass screen. All compounds reduced cell viability in a concentration‐dependent manner (Figure 4A–C, Figure S1). Among the tested series, compounds **5b** and **5f** displayed the most potent cytotoxic activity in three representative breast cancer cell lines (Table 1), exhibiting lower IC_50_ values compared to the remaining analogues. More importantly, **5b** and **5f** showed IC_50_s lower than 5 µM in the triple‐negative breast cancer cell line MDA‐MB‐231; thus, we selected these two compounds for further evaluation.

**TABLE 1:** IC_50_ values of compounds 5a‐5f in breast cancer cell lines.

Compound	IC_50_, µM		
	JIMT1	MDA‐MB‐231	LY2
Fimepinostat	<0.3	<0.3	<0.3
5a	8.58 ± 0.14	5.80 ± 0.60	10.9^a^
5b	5.72 ± 0.76	4.40 ± 0.54	4.68 ± 1.34
5c	10.49^a^	9.43^a^	10.03^a^
5d	8.97 ± 2.64	13.13 ± 6.08	10.34 ± 3.00
5e	15.59 ± 6.48	10.38^a^	8.84 ± 3.47
5f	3.50 ± 0.95	3.30 ± 0.13	8.81 ± 0.59

a The fit did not converge reliably, likely due to incomplete inhibition within the tested range.

To investigate if **5b** and **5f** were acting as multitarget inhibitors**,** we evaluated the modulation of HDAC6 and PI3K signaling pathways by western blot analysis (Figure 4D,E). Treated LY2, JIMT1, and MDA‐MB‐231 cells were lysed after 24 h exposure to **5b** and **5f** at concentrations of 1, 3, and 10 µM. Inhibition of HDAC6 was confirmed by the accumulation of acetylated α‐tubulin at a concentration of 3 µM, further increasing at the highest concentration tested of 10 µM. However, at a concentration of 10 µM, we also started to observe inhibition of class I HDAC, based on the increased levels of acetylation of H3. The evaluation of PI3K pathway modulation was performed through the analysis of decreased phosphorylation of AKT, such as it was observed with the treatment of **5b** and **5f** at 10 µM. Thus, increased acetyl‐α‐tubulin and reduced p‐AKT/AKT ratios are consistent with HDAC6 and PI3K pathway modulation; however, isoform‐specific cellular engagement was not directly established. It is also important to note that there is inhibition of other members of the HDAC family.

Both compounds, **5b** and **5f**, demonstrated potent inhibition of HDAC6, with IC_50_ values in the single‐digit nanomolar range (Table 2). Specifically, compound **5b** exhibited an IC_50_ of 2.33 nM, while compound **5f** showed an IC_50_ of 6.02 nM. This high level of potency suggests that both compounds share similar binding modes. The presence of 3‐hydroxyphenyl and 6‐methoxypyridin‐3‐yl groups, which are likely exposed to the solvent, does not appear to significantly affect the binding mode to HDAC6. For PI3Kα inhibition, structural modifications had a discernible impact on compound potency. The introduction of a 3‐hydroxy group to the phenyl ring in compound **5b** resulted in robust nanomolar inhibition, with an IC_50_ of 17.5 nM. This finding aligns with previous reports indicating that such a modification enhances p110α inhibition [35]. In contrast, the inclusion of a 6‐methoxypyridin‐3‐yl group in compound **5f**, also present in CUDC‐907, resulted in a decrease in the potency, as evidenced by an IC_50_ of 236 nM. Our previous studies with the NAH series corroborated these observations [25]. In those cases, compounds with the 6‐methoxypyridin‐3‐yl group positioned in the affinity pocket also showed approximately a ten‐fold decrease in potency for PI3Kα inhibition when compared to an analogue featuring the 3‐hydroxyphenyl group.

**TABLE 2:** HDAC6 and PI3Kα inhibition data for 5b and 5f.

**Compound**	**IC**_**50**_ **,** **nM** a	
	**HDAC6**	**PI3Kα**
**5b**	2.3	17.5
**5f**	6.0	236
**Trichostatin A**	1.3	N.D
**PI‐103**	N.D	2.8

Abbreviations: N.D. = not determined.

a The assays were conducted by the Reaction Biology Corporation, Malvern, PA. Compounds were tested in the 10‐dose IC_50_ mode with threefold serial dilutions starting at 1 μM.

The inhibitory profiles of **5b** and **5f** were evaluated against selected HDAC isoforms (HDAC1, HDAC3, HDAC4, HDAC8, and HDAC10) and PI3K isoforms (PI3Kδ, PI3Kγ, and PI3Kβ) at a fixed concentration of 0.5 µM (Table 3). Both compounds exhibited notable inhibition of HDAC class I and IIb, while showing weak or negligible inhibition of HDAC4 (class IIa). While we explored the phenyl ring in the linker region to achieve selectivity for the HDAC6 isoform [13, 33], we still observed HDAC class I inhibition. Although **5b** and **5f** were less potent toward HDAC1 inhibition, we speculate that this may be related to the higher flexibility and additional interactions provided by the cap group at the surface of HDACs. A similar profile has been observed for other phenyl‐based hydroxamic acids, which are more potent toward HDAC6 inhibition but also exhibit inhibitory activity against HDAC1 [36, 37]. In the PI3K panel, compounds **5b** and **5f** showed stronger inhibition toward PI3Kδ and less potent inhibition across PI3Kγ and PI3Kβ. It is important to highlight that PI3Kδ is mainly expressed in hematopoietic system and plays roles in leukocyte signaling, T‐cell activation, and immune cell trafficking [38]. FDA‐approved drug idelalisib is a selective PI3Kδ inhibitor in B‐cell malignancies; however, this drug is also related to disruption of the immune system, which can lead to immune manifestations, such as infections [38, 39]. In addition, while isoform‐selective PI3K inhibitors are well tolerated, pan‐inhibition of PI3K is less expected that compensatory mechanisms by the action of other PI3Ks can take place [40]. Thus, a more balanced profile of PI3K inhibition can lead to an overall lack of toxicity of PI3K inhibitors [39]. Collectively, these data support a series of compounds with broader inhibitory activity toward the HDAC and PI3K families. The selectivity profile further corroborates the Western blot analysis, which showed an increase in the acetylation levels of H3.

**TABLE 3:** Selectivity profile 5b and 5f against HDAC and PI3K families.

	**Enzyme % activity (relative to DMSO controls) at 0.5 µM** a	
	**5b**	**5f**
**HDAC1**	35.86	35.68
**HDAC3**	22.59	26.60
**HDAC4**	88.97	98.42
**HDAC8**	46.29	46.88
**HDAC10**	15.91	28.14
**PI3Kβ**	40.13	86.92
**PI3Kδ**	8.24	59.21
**PI3Kγ**	29.16	77.72

a The assays were conducted by the Reaction Biology Corporation, Malvern, PA. Compounds were tested in a single‐dose duplicate mode at 0.5 μM.

To understand the differences between target‐based activity and cell‐based activity, as the compounds showed potent inhibition of HDACs and PI3Ks, we evaluated the physicochemical and ADME properties of **5b** and **5f** and compared them with CUDC‐907. Using DeepPK, a tool for in silico prediction of pharmacokinetic and toxicity properties based on deep learning [41], we determined the predicted permeability (in vitro) of **5b** and **5f**. The predicted values were logP_aap_ = −6.25 cm/s and logP_aap_ = −5.73 cm/s, respectively, while CUDC‐907 showed a predicted value of logP_aap_ = −5.25 cm/s. Thus, CUDC‐907 is predicted to possess higher permeability than **5b** and **5f**. Within the series, **5f** was predicted to be more permeable than **5b**, which corroborates the similar cell‐based activity of **5b** despite its lower potency in the inhibition of HDACs and PI3Ks. In addition, it is important to note that these compounds were less potent in class I HDAC inhibition, which could also contribute to the observed effects on cell viability.

**FIGURE 5:**
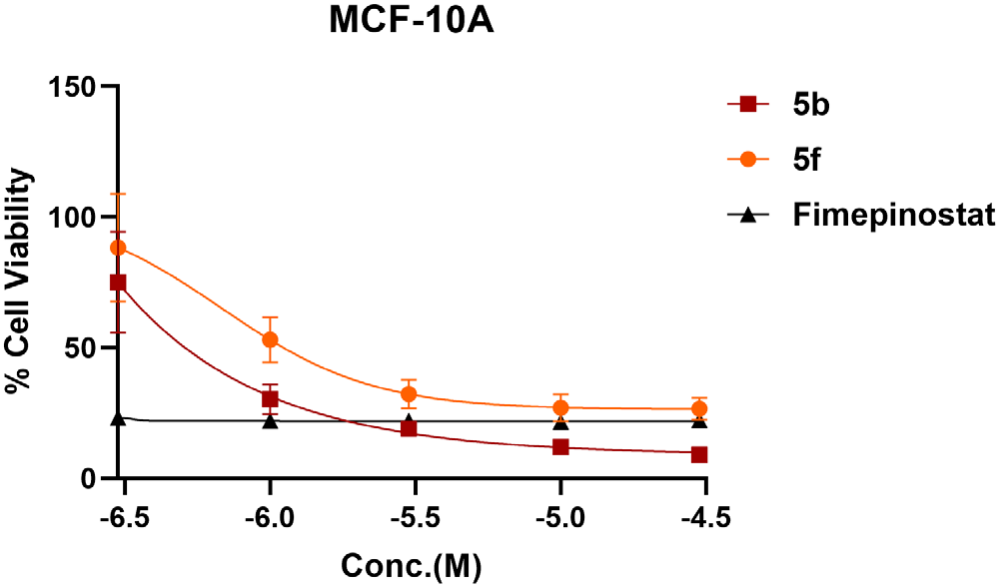
Dose‐dependent effects of compound treatment (**5b** and **5f**) on nontransformed breast cell viability across MCF‐10A.

To further assess the therapeutic window and potential of compounds **5b** and **5f**, we evaluated their effects on cell viability in the nontransformed cell line MCF‐10A, using Fimepinostat as a comparator based on its similar pan‐selectivity profile. Compounds **5b** and **5f** displayed cytotoxic activity against MCF‐10A cells, with IC_50_ values below 1 µM (Figure 5). A similar profile was observed for fimepinostat, which exhibited an IC_50_ below 0.3 µM, most likely due to pan‐inhibition of the PI3K and HDAC families [42]. Thus, further optimization will be required to improve the therapeutic index.

### 2.4 Docking and Molecular Dynamics Simulations

**FIGURE 6:**
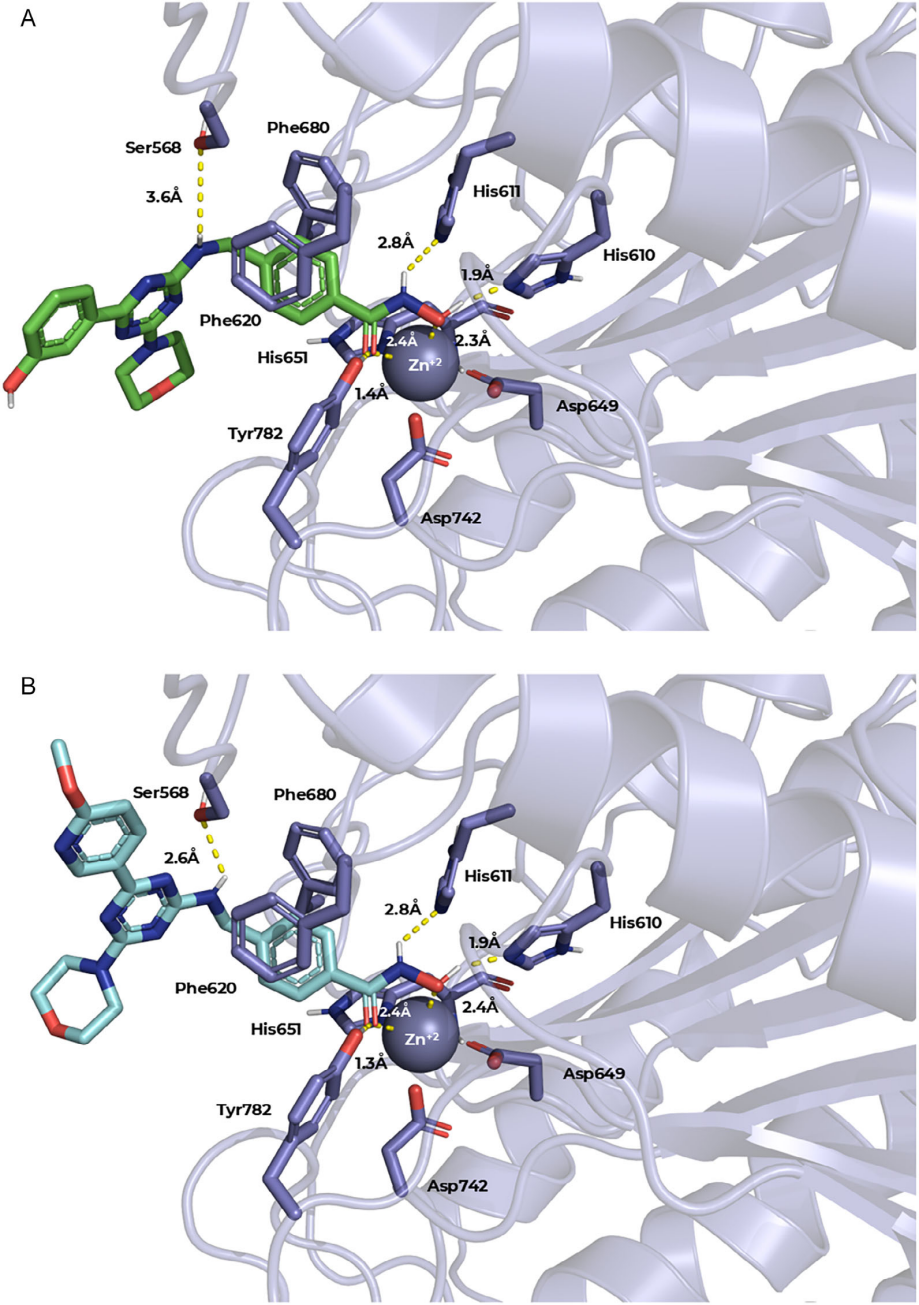
Predicted binding modes of compounds **5b** (LF Δ*G* = −10.9 kcal/mol) and **5f** (LF Δ*G* = −10.5 kcal/mol) within HDAC6 (PDB: 5EDU) obtained from molecular docking analysis. (A) Predicted binding pose of **5b**, with ligand carbons shown in green. (B) Predicted binding pose of **5f**, with ligand carbons shown in cyan.

To understand how **5b** and **5f** interact with HDAC6 and PI3Kα, we performed a molecular docking with these targets. As illustrated in Figure 6, both derivatives **5b** and **5f** displayed comparable binding modes toward HDAC6 (PDB: 5EDU) [43]. In both cases, the ligands chelate the catalytic Zn^2+^ ion in a bidentate fashion, maintaining coordination distances in the range of 2.3–2.4 Å. Additionally, they establish hydrogen bonds with the catalytic residues His610, His611, and Tyr782. Regarding the Ser568 residue, which is highlighted for contributing to the gain of selectivity toward isoform 6 [13, 44], it can be observed that **5f** maintained a distance of 2.6 Å, consistent with a plausible hydrogen bond. In contrast, such interaction was not retained in the case of **5b**, for which the distance increased to approximately 3.6 Å. Moreover, both ligands adopt a favorable orientation of the phenyl spacer between Phe620 and Phe680, enabling π–π stacking interactions within the enzyme channel.

**FIGURE 7:**
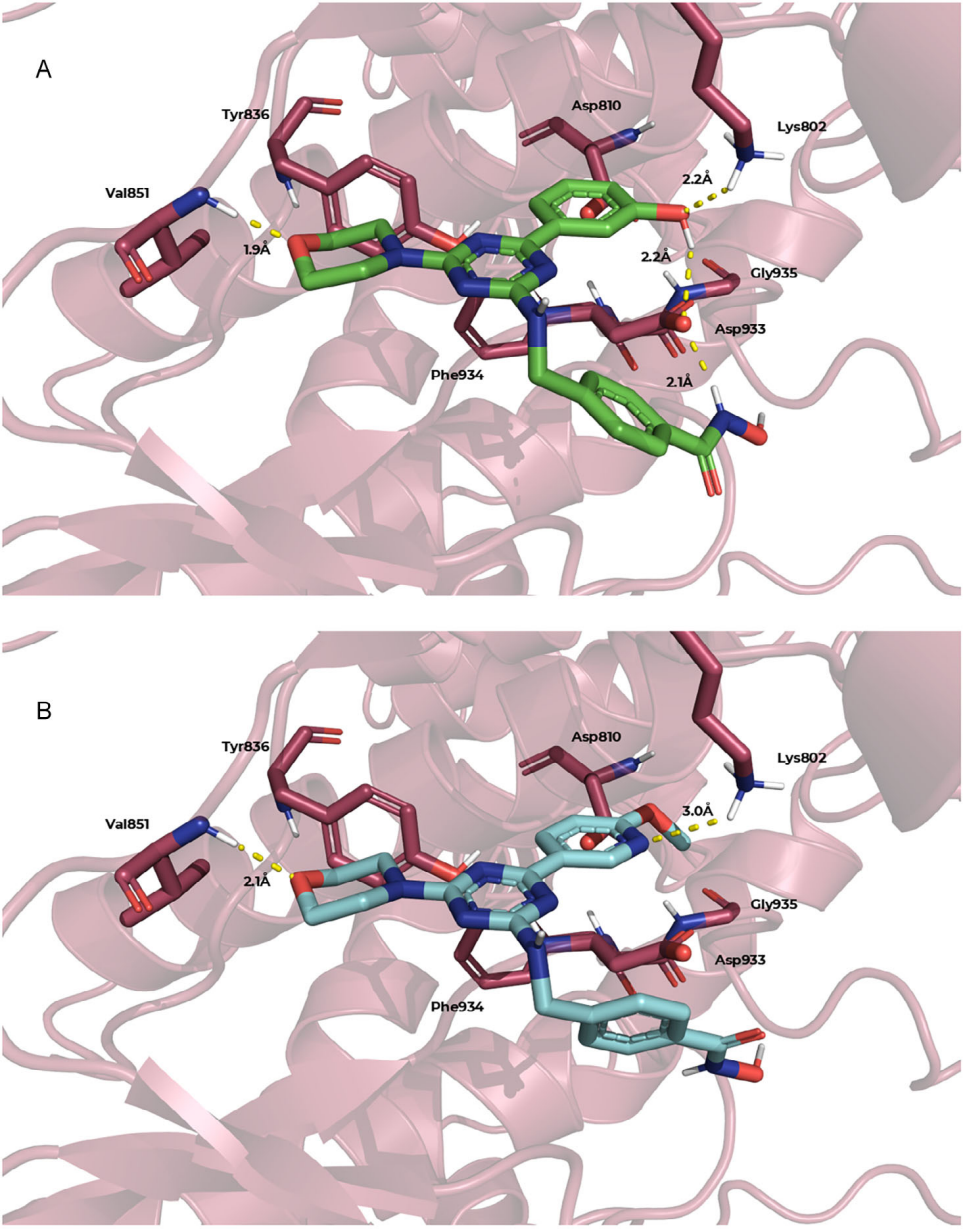
Predicted binding modes of compounds **5b** (LF Δ*G* = −9.2 kcal/mol) and **5f** (LF Δ*G* = −8.8 kcal/mol) within PI3K (PDB: 4L23) obtained from molecular docking analysis. (A) Predicted binding pose of **5b**, with ligand carbons shown in green. (B) Predicted binding pose of **5f**, with ligand carbons shown in cyan.

The protocol applied to PI3Kα (PDB: 4L23) [45] followed the same methodology as that employed for HDAC6. Figure 7 depicts the binding poses of compounds **5b** and **5f** within the PI3Kα, revealing a hydrogen bond between the morpholine moiety of the ligands and the backbone of Val851, which constitutes part of the hinge region and is pharmacophoric for PI3Kα inhibition [27], with a distance of 1.9–2.1 Å. An additional interaction with the catalytic Lys802, located within the affinity pocket, was also observed. For **5b**, this interaction was mediated by the phenolic oxygen atom, whereas for **5f,** the hydrogen bond was established through the pyridine nitrogen, while the methoxy group remained oriented away from Lys802 in the docking pose. Moreover, compound **5b** showed contacts between its phenolic and hydroxamic acid moieties and Asp933, the aspartate residue of the aspartate‐phenylalanine‐glycine (DFG) motif. However, the observed geometry suggests that these contacts are weak and unlikely to represent relevant hydrogen bonds. Compound **5f** did not display a similar interaction pattern in this region.

Molecular dynamics simulations were performed for the HDAC6–ligand complexes obtained from molecular docking, as well as for the apo enzyme, to assess the stability of the systems and the persistence of key interactions over time. From a force‐field perspective, ligand parameters were assigned using OpenFF 2.2.0 (Sage) in combination with AM1‐BCC charges, a widely adopted small‐molecule parameterization strategy trained and benchmarked against quantun mechanics (QM) reference data and commonly used in protein–ligand MD with assisted model building and energy refinement (AMBER) biomolecular force fields [46, 47]. The catalytic Zn^2+^ site was described using an AMBER‐compatible transferable classical nonbonded representation, an approach that has been extensively discussed and validated for Zn^2+^ in metalloproteins and in protein–ligand contexts [48, 49, 50].

**FIGURE 8:**
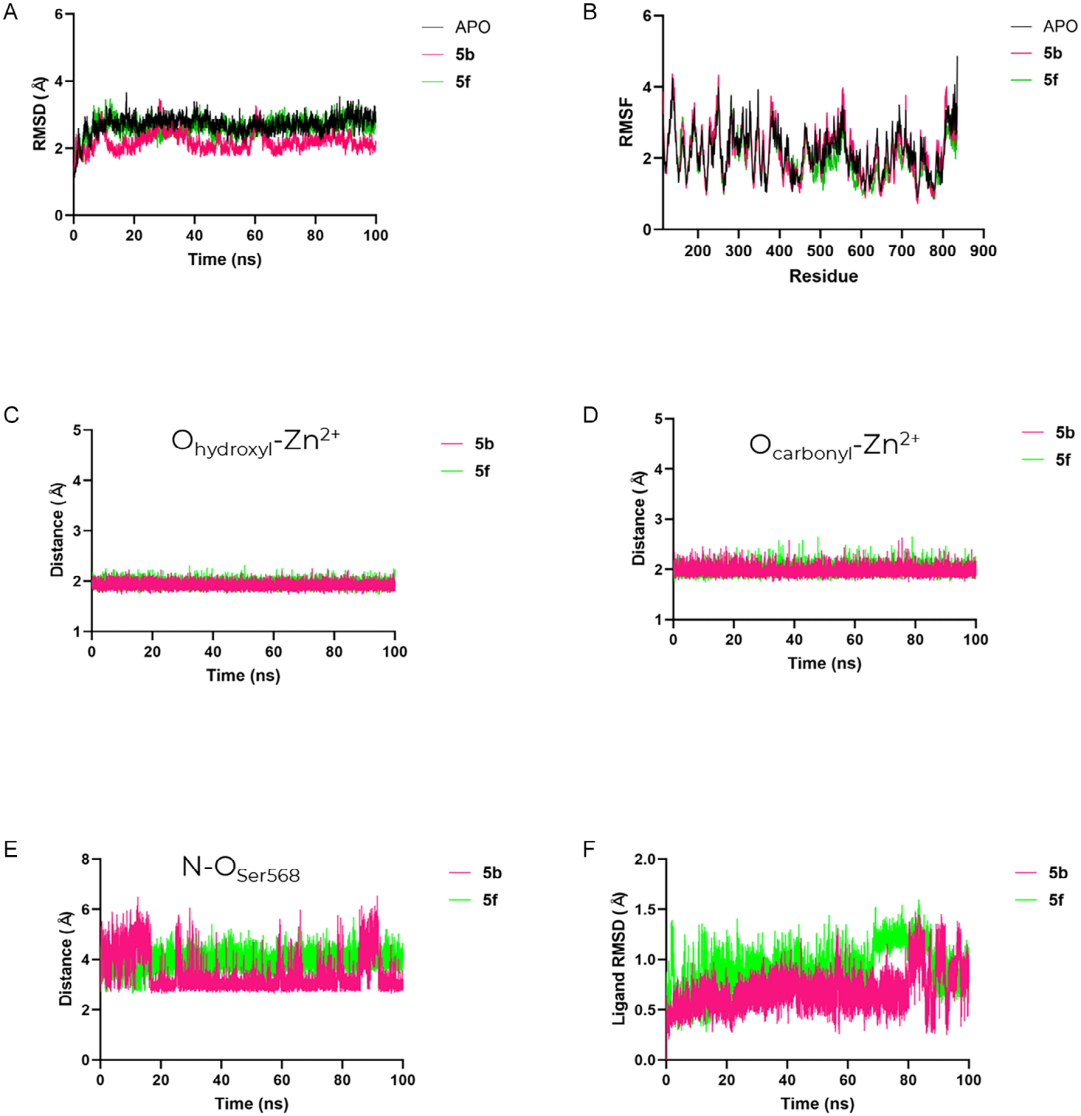
Molecular dynamics analysis of HDAC6 in the apo form and in complex with compounds **5b** and **5f**. (A) Total root‐mean‐square deviation (RMSD) along the simulation, showing overall conformational stability of both apo and complexed systems. (B) Root‐mean‐square fluctuation (RMSF) per residue, indicating minor local flexibility changes upon ligand binding. (C,D) Time evolution of the distances between the Zn^2+^ ion and the coordinating atoms of the ligands, evidencing a persistent bidentate coordination around 2 Å. (E) Distance between residue Ser568 and the ligands, revealing transient interaction for **5b** and weaker persistence for **5f**. (F) Ligand root‐mean‐square deviation (RMSD) values, remaining below 1.5 Å throughout the trajectory, with most fluctuations associated with the solvent‐exposed cap group.

As shown in Figure 8A, the root‐mean‐square deviation (RMSD) profiles indicate that all systems, both the apoprotein and those complexed with ligands, remained conformationally stable throughout the simulation, displaying only minor deviations from the initial structures. This behavior is consistent with expectations, since the crystallographic structure used as a reference already contains a bound inhibitor with structural similarity to the tested compounds. The root‐mean‐square fluctuation (RMSF) analysis (Figure 8B) corroborates this observation, revealing minimal residue fluctuations and suggesting that ligand binding exerts little influence on the overall protein flexibility. Importantly, an internal consistency check for the Zn^2+^–hydroxamate description was obtained by monitoring metal coordination distances: further examination of the metal coordination distances (Figures 8C and D) confirmed a persistent bidentate coordination of the ligands to the catalytic Zn^2+^ ion, maintaining an average distance of approximately 2 Å along the trajectory. The interaction with residue Ser568 exhibited different behaviors between the two ligands: for compound **5f**, the average distance remained near 4 Å, whereas for **5b**, the interaction was slightly stronger, averaging around 3 Å but showing a transient profile (Figure 8E). Finally, the ligand RMSD values (Figure 8F) remained below 1.5 Å for both compounds, indicating that the molecules remained well accommodated within the binding pocket. Most of the observed fluctuations were associated with the solvent‐exposed cap region, reflecting its expected higher mobility.

**FIGURE 9:**
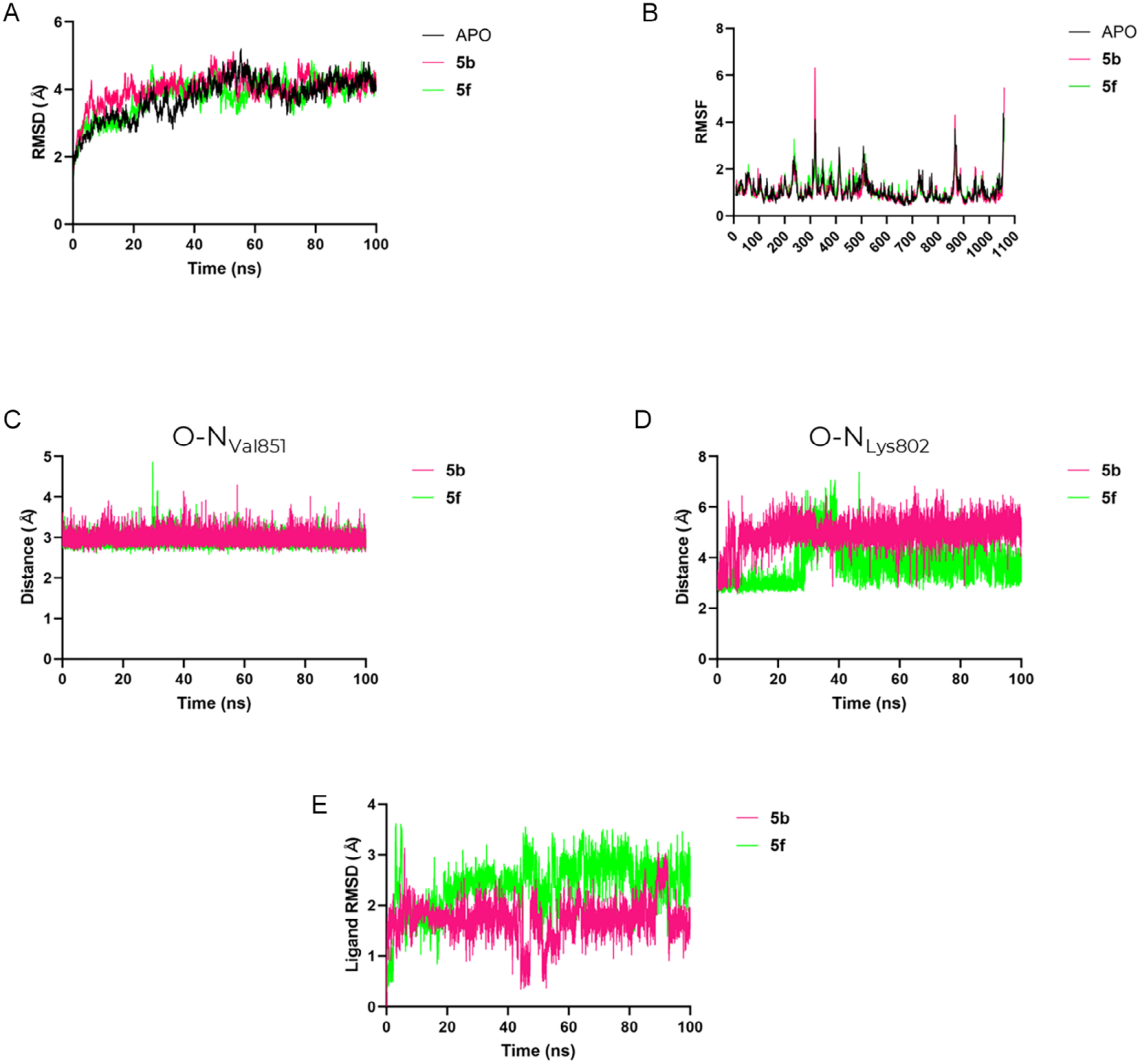
Molecular dynamics analyses of PI3Kα in the apo form and in complex with compounds **5b** and **5f**. (A) Total root‐mean‐square deviation (RMSD) of the system over the simulation time, showing comparable structural stability for both apo and ligand‐bound states. (B) Root‐mean‐square fluctuation (RMSF) per residue, indicating minor local fluctuations and preservation of the overall enzyme flexibility upon ligand binding. (C) Time evolution of the distance between the oxygen atom of the morpholine ring and the backbone nitrogen of Val851, consistent with a stable hydrogen bond for both ligands. (D) Distance between the ligand and the side‐chain nitrogen of the catalytic Lys802, showing a weaker and more variable interaction for the phenolic hydrogen of **5b** and a closer, more persistent contact for the pyridine nitrogen of **5f**. (E) Ligand RMSD values ranging from 0.5 to 1.2 Å, confirming high positional stability within the binding pocket throughout the trajectory.

For the PI3Kα systems, a comparable stability pattern was observed. As shown in Figure 9A, the total RMSD values of the system remained within the same range for both the apo form and the ligand‐bound complexes, indicating that ligand association did not induce significant conformational deviations over the course of the simulation. This observation is supported by the RMSF analysis (Figure 9B), which reveals only minor local fluctuations, suggesting that the overall flexibility of the enzyme was largely preserved upon ligand binding. Analysis of key intermolecular distances provided additional insights into the nature of ligand–protein interactions. As shown in Figure 9C, the distance between the oxygen atom of the morpholine ring and the backbone nitrogen of Val851 remained consistent with a stable hydrogen bond for both ligands throughout the simulation. In contrast, Figure 9D shows that the distance between the ligand and the side‐chain nitrogen of the catalytic Lys802 fluctuated between 4 and 6 Å for the phenolic oxygen of **5b**, whereas **5f** showed a shift from what was observed in the molecular docking analysis, presenting a hydrogen bond through the methoxy group, which maintained a closer and more persistent interaction, averaging between 3 and 4 Å along most of the trajectory. In addition, the initially observed hydrogen bond between the pyridine nitrogen atom of **5f** was lost after 20 ns, indicating that it was not as stable as the docking analysis predicted. Finally, the ligand RMSD profiles (Figure 9E) ranged from 0.5 to 1.2 Å, indicating minimal structural displacement within the binding pocket. These results confirm that both ligands remained well accommodated and dynamically stable during the simulation, preserving the key interactions identified in the docking analysis.

## 3 Conclusions

In summary, we proposed structural modifications to LASSBio‐2208 (**1**) to design a novel series of triazine‐based dual HDAC/PI3K inhibitors. Among these, compounds **5b** and **5f** exhibited the most promising biological profiles, demonstrating antiproliferative activity in breast cancer cell lines and equipotent inhibition of HDAC6 and PI3Kα in target‐based assays. NMR analysis revealed the presence of conformers in DMSO‐*d*_6_ solution, although this phenomenon was not further investigated in water. Importantly, conformational behavior did not appear to affect target engagement. Both compounds displayed low nanomolar IC_50_ values against HDAC6 and PI3Kα, confirming their strong biochemical potency, showing a promising profile of inhibition of these two targets, similar to the inhibition data from LASSBio‐2208. Thus, our designed approach was successful. However, in cell‐based assays, concentrations in the micromolar range (3–10 µM) were required to achieve comparable inhibitory effects, suggesting limited cell permeability. In addition, while they showed a promising profile for inhibition of HDAC6 and PI3Kα, they were also shown to inhibit other members of the HDAC and PI3K family, probably due to an increased flexibility of this series of compounds. Thus, in order to achieve higher selectivity toward HDAC6 isoform, we expect that modifications of the ZBG can lead to compounds with increased selectivity by exploring mercaptoacetamides [51] or difluoromethyl‐1,3,4‐oxadiazole (DFMO) [52, 53, 54] systems. Thus, **5b** and **5f** can be considered promising lead compounds and valuable starting points for further structural optimization.

## 4 Methods

### 4.1 General Information

^1^H‐NMR spectra were determined in deuterated dimethyl sulfoxide or deuterated chloroform using a Bruker AVANCE 400 at 400 MHz. ^13^C‐NMR spectra were resolved using the same spectrometers at 100 MHz and using the same solvents. All spectra were analyzed using MestReNova software, and the residual undeuterated solvent signals were used as internal references. The progress of all the reactions was monitored through thin‐layer chromatography performed on 2.0 × 6.0 cm^2^ aluminum sheets precoated with silica gel 60 (HF‐254, Merck) to a thickness of 0.25 mm. The developed chromatograms were viewed under ultraviolet light (254−365 nm) and treated with iodine vapor. Flash column chromatography was carried out using silica gel, particle size 0.04–0.063 mm. The reagents and solvents were purchased from commercial suppliers and used as received. High‐resolution electrospray ionization (ESI) mass spectrometry was carried out in positive mode on a Bruker CompactTM mass spectrometer. The samples were prepared using 1 mL of a mixture of acetonitrile and water (0.1% formic acid) (1:1). The HRMS was performed by Dr. Gary Hessman from the School of Chemistry, Trinity College Dublin, Ireland. The spectra were analyzed in Bruker Compass DataAnalysis 4.1. The high‐performance liquid chromatography was performed with a Shimadzu SIL‐20AHT HPLC instrument equipped with a Shimadzu SPD‐20AV prominence UV/Vis detector using a Kromasil 100–5 C18 column (4.6 × 250 mm). The mobile phase used was acetonitrile and water (0.1% TFA) in a 1:1 mixture. The isocratic HPLC mode was used, and the flow rate was 1.0 mL/min. The purity of the compounds was higher than 95% using 280 nm as the wavelength.

### 4.2 Synthesis of 4‐(4,6‐Dichloro‐1,3,5‐triazin‐2‐yl)Morpholine (2)

In a round‐bottom flask, 2.0 g (10.85 mmol) of cyanuric chloride (**1**) and 100 mL of THF were added, and the flask was placed in an ice bath. A solution containing 50 mL of THF, 984 µL (11.42 mmol) of morpholine, and 1.91 mL (13.7 mmol) of triethylamine was prepared and transferred to an addition funnel. This solution was added dropwise to the round‐bottom flask while maintaining the mixture in the ice bath. The reaction mixture was stirred for 30 min, and progress was monitored by thin‐layer chromatography (TLC). Afterward, the solvent was partially removed under reduced pressure. The resulting solution was poured into 50 mL of deionized water, leading to the precipitation of a white solid. The precipitate was collected by filtration, yielding 1.84 g of 4‐(4,6‐dichloro‐1,3,5‐triazin‐2‐yl)morpholine (**2**), corresponding to a 73% yield. ^1^H NMR (400 MHz, CDCl_3_) δ 3.85 – 3.79 (m, 4H), 3.72 – 3.66 (m, 4H). ^13^C NMR (100 MHz, CDCl_3_) δ 170.6, 164.2, 66.5, 44.6.

### 4.3 Synthesis of Methyl 4‐(((4‐Chloro‐6‐morpholino‐1,3,5‐triazin‐2‐yl)Amino)Methyl)Benzoate (3)

In a round‐bottom flask, 2.0 g (8.51 mmol) of 4‐(4,6‐dichloro‐1,3,5‐triazin‐2‐yl)morpholine (**2**) and 100 mL of THF were added, and the flask was placed in an ice bath. A solution containing 100 mL of THF, 1.81 g (8.96 mmol) of methyl 4‐(aminomethyl)benzoate hydrochloride, 3.12 mL (22.39 mmol) of triethylamine, and 10 mL of deionized water was prepared and transferred to an addition funnel. This solution was added dropwise to the round‐bottom flask while maintaining the mixture in the ice bath. The reaction mixture was stirred overnight at room temperature. The solvent was partially removed under reduced pressure. The resulting solution was poured into 50 mL of deionized water, leading to the precipitation of a white solid. The precipitate was collected by filtration, yielding 1.84 g of methyl 4‐(((4‐chloro‐6‐morpholino‐1,3,5‐triazin‐2‐yl)amino)methyl)benzoate (**3**) as a white powder, corresponding to a 60% yield. ^1^H NMR (400 MHz, CDCl_3_) δ 7.92 (d, *J* = 8.2 Hz, 2H), 7.29 (d, *J* = 8.1 Hz, 2H), 6.97 (t, *J* = 6.0 Hz, 1H), 4.62 and 4.59 (2d, *J* = 6.1 Hz, 2H), 3.84 (s, 3H), 3.75 – 3.52 (m, 8H). ^13^C NMR (100 MHz, CDCl_3_) δ 169.0, 166.8, 165.7, 164.4, 143.8, 129.9, 127.1, 66.6, 66.4, 52.1, 44.6, 44.0, 43.9.

## 5 General Procedure for the Suzuki Coupling Step (4a‐4f)

In a small round‐bottom flask, 400 mg (1.1 mmol) of 4‐chloro‐6‐(4‐morpholinyl)‐1,3,5‐triazin‐2‐amine (**3**), 232.9 mg (2.2 mmol) of sodium carbonate, and 2.2 mmol of the respective boronic acid were combined with 10 mL of THF, 10 mL of *t*‐BuOH, and 2 mL of water. Finally, 38.4 mg (5 mol%) of palladium (II) dichloride bis(triphenylphosphine) [PdCl_2_(PPh_3_)_2_] was added. The reaction mixture was heated at 90°C with stirring for 16 h. After completion, the mixture was cooled to room temperature and poured into 50 mL of water. The product was extracted with ethyl acetate, and the combined organic layers were washed with brine and dried over sodium sulfate. The solvent was removed under reduced pressure, and the crude product was purified by column chromatography.

### 5.1 Methyl 4‐(((4‐Morpholino‐6‐Phenyl‐1,3,5‐Triazin‐2‐yl)Amino)Methyl)Benzoate (4a)

304 mg of the title compound was obtained as a white powder, corresponding to a 68% yield. ^1^H NMR (400 MHz, DMSO‐*d*_6_) δ 8.34 – 8.24 (m, 2H), 8.18, 8.02 (2t, *J* = 6.3 Hz, 1H), 7.92 (d, *J* = 8.3 Hz, 2H), 7.57 – 7.40 (m, 5H), 4.69, 4.57 (2d, *J* = 6.2 Hz, 1H), 3.96 – 3.50 (m, 11H). ^13^C NMR (100 MHz, DMSO) δ 169.5, 166.1, 166.0, 165.9, 164.6, 146.2, 145.8, 136.8, 131.5, 131.4, 129.2, 129.2, 128.2, 128.0, 127.9, 127.8, 127.6, 127.4, 65.9, 52.0, 43.5, 43.2.

### 5.2 Methyl 4‐(((4‐(3‐Hydroxyphenyl)‐6‐Morpholino‐1,3,5‐Triazin‐2‐yl)Amino)Methyl)Benzoate (4b)

240 mg of the title compound was obtained as a white powder, corresponding to a 51% yield. ^1^H NMR (400 MHz, DMSO) δ 9.54, 9.55 (2s, 1H), 8.12, 7.98 (2t, *J* = 6.2 Hz, 1H), 7.92 (d, *J* = 8.1 Hz, 2H), 7.79 – 7.69 (m, 2H), 7.50, 7.48 (2d, *J* = 8.2 Hz, 2H), 7.31 – 7.18 (m, 1H), 6.97 – 6.84 (m, 1H), 4.69, 4.57 (2d, *J* = 6.2 Hz, 2H), 3.90 – 3.51 (m, 11H). ^13^C NMR (100 MHz, DMSO) δ 170.0, 166.6, 166.5, 166.3, 165.0, 157.7, 157.6, 146.6, 146.3, 138.7, 130.0, 129.6, 128.5, 128.0, 127.9, 119.3, 119.2, 118.9, 118.8, 115.1, 66.4, 66.3, 52.5, 43.9, 43.7.

### 5.3 Methyl 4‐(((4‐Morpholino‐6‐(Pyridin‐3‐yl)−1,3,5‐Triazin‐2‐yl)Amino)Methyl)Benzoate (4c)

336 mg of the title compound was obtained as a white powder, corresponding to a 75% yield. ^1^H NMR (400 MHz, DMSO) δ 9.38 (d, *J* = 14.5 Hz, 1H), 8.76 – 8.65 (m, 1H), 8.54 (t, *J* = 7.0 Hz, 1H), 8.29, 8.13 (2t, *J* = 6.1 Hz, 1H), 7.91 (d, *J* = 8.0 Hz, 2H), 7.55 – 7.51 (m, 1H), 7.50 – 7.45 (m, 2H), 4.68, 4.57 (2d, *J* = 6.1 Hz, 2H), 3.88 – 3.54 (m, 11H). ^13^C NMR (100 MHz, DMSO) δ 168.6, 166.6, 166.3, 166.1, 164.8, 152.4, 149.7, 149.6, 146.5, 146.1, 135.8, 135.7, 132.6, 132.5, 129.7, 129.6, 128.5, 128.4, 128.0, 127.8, 124.0, 66.4, 52.5, 43.9, 43.7.

### 5.4 Methyl 4‐(((4‐(6‐Aminopyridin‐3‐yl)‐6‐Morpholino‐1,3,5‐Triazin‐2‐yl)Amino)Methyl)Benzoate (4d)

320 mg of the title compound was obtained as a yellow powder, corresponding to a 69% yield. ^1^H NMR (400 MHz, DMSO) δ 8.86, 8.81 (2s, 1H), 8.21 – 8.10 (m, 1H), 8.00, 7.86 (2t, *J* = 6.2 Hz, 1H), 7.91 (d, *J* = 8.2 Hz, 2H), 7.86 (t, *J* = 6.5 Hz, 1H), 7.48, 7.46 (2d, *J* = 6.8 Hz, 2H), 6.53 (s, 2H), 6.49 – 6.40 (m, 1H), 4.64, 4.65 (2d, *J* = 6.1 Hz, 2H), 3.88 – 3.51 (m, 11H). ^13^C NMR (100 MHz, DMSO) δ 169.1, 166.6, 166.1, 166.0, 164.8, 162.2, 150.1, 149.8, 148.1, 146.8, 146.5, 137.4, 137.0, 129.6, 128.4, 128.0, 127.8, 120.7, 107.4, 66.5, 66.4, 52.5, 43.8, 43.6.

### 5.5 Methyl 4‐(((4‐(2‐Aminopyrimidin‐5‐yl)‐6‐Morpholino‐1,3,5‐Triazin‐2‐yl)Amino)Methyl)Benzoate (4e)

380 mg of the title compound was obtained as a yellow powder, corresponding to an 81% yield. ^1^H NMR (400 MHz, DMSO) δ 9.01, 8.96 (2s, 2H), 8.10, 7.96 (2t, *J* = 6.2 Hz, 1H), 7.91 (d, *J* = 8.2 Hz, 2H), 7.48, 7.46 (2d, *J* = 7.0 Hz, 2H), 7.26 (s, 2H), 4.64, 4.55 (2d, *J* = 6.1 Hz, 2H), 3.85 – 3.56 (m, 11H). ^13^C NMR (100 MHz, DMSO) δ 167.7, 166.6, 166.0, 165.9, 165.2, 164.6, 159.0, 158.8, 158.5, 146.7, 146.3, 129.6, 128.4, 128.4, 128.0, 127.8, 119.0, 66.5, 52.5, 43.8, 43.6.

### 5.6 Methyl 4‐(((4‐(6‐Methoxypyridin‐3‐yl)‐6‐Morpholino‐1,3,5‐Triazin‐2‐yl)Amino)Methyl)Benzoate (4f)

297 mg of the title compound was obtained as a white powder, corresponding to a 62% yield. ^1^H NMR (400 MHz, DMSO) δ 9.05, 9.02 (2d, *J* = 1.8 Hz, 1H), 8.53 – 8.38 (m, 1H), 8.18, 8.04 (2t, *J* = 6.2 Hz, 1H), 7.92 (d, *J* = 8.2 Hz, 2H), 7.49, 7.47 (2d, *J* = 8.0 Hz, 2H), 6.90, 6.86 (2d, *J* = 8.7 Hz, 1H), 4.67, 4.56 (2d, *J* = 6.1 Hz, 2H), 3.92, 3.90 (2s, 3H), 3.87 – 3.53 (m, 11H). ^13^C NMR (100 MHz, DMSO) δ 168.4, 166.6, 166.2, 166.0, 166.0, 164.8, 164.8, 148.3, 148.1, 146.6, 146.2, 138.9, 129.7, 129.6, 128.5, 128.4, 128.0, 127.8, 126.5, 110.6, 66.4, 54.1, 52.5, 43.9, 43.7.

## 6 General Procedure for the Synthesis of Hydroxamic Acids (5a‐5f)

The respective methyl ester (**4a**‐**4f**) (0.6 mmol) was dissolved in 10 mL of THF, and hydroxylamine (50% in water, 6.1 mL, 100 mmol, 166 eq.) was added, followed by potassium hydroxide in methanol (4 M, 12.5 mL, 83 mmol). The resulting mixture was stirred at room temperature for 10 min, and progress was monitored by TLC. The reaction mixture was poured into 10 mL of water, and the pH was adjusted to 7 using acetic acid. The product was extracted with ethyl acetate, and the combined organic layers were washed with 10 mL of saturated sodium bicarbonate solution, followed by 10 mL of saturated brine solution. The organic phase was dried over sodium sulfate, and the solvent was partially removed under reduced pressure. Before complete solvent removal, the product was precipitated with diethyl ether and collected by filtration.

### 6.1 N‐Hydroxy‐4‐(((4‐Morpholino‐6‐Phenyl‐1,3,5‐Triazin‐2‐yl)Amino)Methyl)Benzamide (5a)

170 mg of the title compound was obtained as a white powder, corresponding to a 70% yield. ^1^H NMR (400 MHz, DMSO) δ 11.17 (s, 1H), 9.02 (s, 1H), 8.30 (d, *J* = 7.1 Hz, 2H), 8.16, 8.00 (2t, *J* = 6.3 Hz, 1H), 7.70 (d, *J* = 8.2 Hz, 2H), 7.57 – 7.37 (m, 5H), 4.66, 4.54 (2d, *J* = 6.2 Hz, 1H), 3.95 – 3.52 (m, 8H). ^13^C NMR (100 MHz, DMSO) δ 169.5, 166.0, 165.9, 164.6, 164.2, 143.7, 143.4, 136.8, 131.5, 131.4, 131.2, 128.2, 128.0, 127.8, 127.3, 127.1, 126.9, 66.0, 43.4, 43.23. HRMS calculated for C_21_H_23_N_6_O_3_: [M+H]^+^ = 407.1832. Found: 407.1826. HPLC purity of 99%.

### 6.2 N‐Hydroxy‐4‐(((4‐(3‐Hydroxyphenyl)‐6‐Morpholino‐1,3,5‐Triazin‐2‐yl)Amino)Methyl)Benzamide (5b)

95 mg of the title compound was obtained as a white powder, corresponding to a 38% yield. ^1^H NMR (400 MHz, DMSO) δ 11.15 (s, 1H), 9.53 (s, 1H), 8.99 (s, 1H), 8.07, 7.93 (2t, *J* = 6.3 Hz, 1H), 7.80 – 7.65 (m, 4H), 7.43, 7.40 (2d, *J* = 8.5 Hz, 2H), 7.25 (td, *J* = 8.1, 3.7 Hz, 1H), 6.91 (d, *J* = 7.3 Hz, 1H), 4.64, 4.53 (2d, *J* = 6.1 Hz, 2H), 3.97 – 3.55 (m, 8H). ^13^C NMR (100 MHz, DMSO) δ 170.0, 166.5, 166.3, 165.1, 164.6, 157.7, 143.9, 138.7, 131.7, 129.6, 127.7, 127.6, 127.3, 119.3, 119.2, 118.8, 115.1, 66.4, 43.8, 43.7. HRMS calculated for C_21_H_23_N_6_O_4_: [M+H]^+^ = 423.1781. Found: 423.1780. HPLC purity of 97.46%.

### 6.3 N‐Hydroxy‐4‐(((4‐Morpholino‐6‐(pyridin‐3‐yl)−1,3,5‐Triazin‐2‐yl)Amino)Methyl)Benzamide (5c)

140 mg of the title compound was obtained as a white powder, corresponding to a 57% yield. ^1^H NMR (400 MHz, DMSO) δ 11.18 (s, 1H), 9.40 (s, 1H), 9.02 (s, 1H), 8.77 – 8.66 (m, 1H), 8.55 (dd, *J* = 7.9, 1.8 Hz, 1H), 8.27, 8.11 (2t, *J* = 6.2 Hz, 1H), 7.70 (d, *J* = 8.2 Hz, 2H), 7.53 – 7.46 (m, 1H), 7.43, 7.41 (2d, *J* = 8.2 Hz, 2H), 4.65, 4.54 (2d, *J* = 6.2 Hz, 2H), 3.95 – 3.55 (m, 8H). ^13^C NMR (100 MHz, DMSO) δ 168.6, 168.5, 166.4, 166.2, 164.8, 164.6, 152.5, 152.4, 149.7, 149.6, 144.0, 143.7, 135.8, 135.7, 132.6, 131.7, 127.8, 127.6, 127.3, 124.0, 66.4, 43.8. HRMS calculated for C_20_H_22_N_7_O_3_: [M+H]^+^ = 408.1784. Found: 408.1783. HPLC purity of 99%.

### 6.4 4‐(((4‐(6‐Aminopyridin‐3‐yl)‐6‐Morpholino‐1,3,5‐Triazin‐2‐yl)Amino)Methyl)‐N‐Hydroxybenzamide (5d)

130 mg of the title compound was obtained as a white powder, corresponding to a 51% yield. ^1^H NMR (400 MHz, DMSO) δ 11.17 (s, 1H), 9.02 (s, 1H), 8.91 – 8.80 (m, 1H), 8.16 (dd, *J* = 8.7, 1.5 Hz, 1H), 7.97, 7.83 (2t, *J* = 6.2 Hz, 1H), 7.69 (d, *J* = 8.2 Hz, 2H), 7.41, 7.39 (2d, *J* = 7.5 Hz, 2H), 6.53 (s, 2H), 6.45 (dd, *J* = 8.6, 5.7 Hz, 1H), 4.60, 4.50 (2d, *J* = 6.1 Hz, 2H), 3.90 – 3.52 (m, 8H). ^13^C NMR (100 MHz, DMSO) δ 169.1, 166.6, 166.1, 166.0, 164.8, 162.2, 150.1, 149.8, 148.1, 146.8, 146.5, 137.4, 137.0, 129.6, 128.4, 128.0, 127.8, 120.7, 107.4, 66.5, 66.4, 52.5, 43.8, 43.6. HRMS calculated for C_20_H_23_N_8_O_3_: [M+H]^+^ = 423.1893. Found: 423.1897. HPLC purity of 99.8%.

### 6.5 4‐(((4‐(2‐Aminopyrimidin‐5‐yl)‐6‐Morpholino‐1,3,5‐Triazin‐2‐yl)Amino)Methyl)‐N‐Hydroxybenzamide (5e)

206 mg of the title compound was obtained as a white powder, corresponding to a 81% yield. ^1^H NMR (400 MHz, DMSO) δ 11.17 (s, 1H), 9.02 (s, 1H), 9.00, 8.98 (2s, 2H), 8.07, 7.93 (2t, *J* = 6.2 Hz, 1H), 7.69 (d, *J* = 8.2 Hz, 2H), 7.41, 7.39 (2d, *J* = 7.7 Hz, 2H), 7.25 (s, 2H), 4.60, 4.51 (2d, *J* = 6.1 Hz, 2H), 3.94 – 3.51 (m, 8H). ^13^C NMR (100 MHz, DMSO) δ 167.7, 167.7, 166.0, 165.8, 165.2, 165.2, 164.6, 164.6, 159.0, 158.8, 144.2, 143.8, 131.6, 127.7, 127.6, 127.3, 119.0, 66.4, 43.7, 43.6. HRMS calculated for C_19_H_22_N_9_O_3_: [M+H]^+^ = 424.1846. Found: 424.1843. HPLC purity of 98.52%.

### 6.6 N‐Hydroxy‐4‐(((4‐(6‐Methoxypyridin‐3‐yl)‐6‐Morpholino‐1,3,5‐Triazin‐2‐yl)Amino)Methyl)Benzamide (5f)

233 mg of the title compound was obtained as a white powder, corresponding to an 89% yield. ^1^H NMR (400 MHz, DMSO) δ 11.17 (s, 1H), 9.05 (d, *J* = 2.1 Hz, 1H), 9.01 (s, 1H), 8.55 – 8.37 (m, 1H), 8.16, 8.01 (2t, *J* = 6.3 Hz, 1H), 7.70 (d, *J* = 8.2 Hz, 2H), 7.42, 7.40 (2d, *J* = 8.4 Hz, 2H), 6.89 (t, *J* = 8.8 Hz, 1H), 4.63, 4.52 (2d, *J* = 6.2 Hz, 2H), 3.92, 3.91 (2s, 3H), 3.88 – 3.55 (m, 8H). ^13^C NMR (100 MHz, DMSO) δ 168.4, 166.2, 166.1, 166.0, 164.8, 164.6, 148.3, 148.1, 144.1, 143.8, 138.9, 138.9, 131.7, 127.8, 127.6, 127.3, 126.5, 110.6, 66.4, 54.1, 43.8. HRMS calculated for C_21_H_24_N_7_O_4_: [M+H]^+^ = 438.1890. Found: 438.1894. HPLC purity of 99%.

### 6.7 Quantum Mechanical Calculations

A density functional theory (DFT) study was initially performed to investigate the possible origin of the duplicated NMR signals observed for the spacer (aminomethyl) region. A tautomeric stability analysis was conducted by generating all plausible tautomeric forms of compound 5a, followed by full geometry optimization at the ωB97XD/6‐31+G(d,p) level of theory, including solvent effects described with the Spartan’24 continuum solvation model (C‐PCM) using the “polar organic solvents” option (*∈* = 37), i.e., a generic polar aprotic medium. The relative energies were used to calculate Boltzmann population distributions, which indicated the predominance of a single tautomeric form. Subsequently, a relaxed PES scan was performed to investigate conformational preferences and the rotational barrier of the bond connecting the spacer amino group to the triazine ring. The dihedral angle N–C–N–H was varied from 0° to 180° in 15° increments, allowing full geometry optimization at each step, using the same ωB97XD/6‐31+G(d,p) level of theory with the same C‐PCM “polar organic solvents” solvation setting (*∈* = 37). All calculations were carried out using the Spartan’24 software. The tautomeric stability and PES analyses described above were performed exclusively for compound 5a, which was used as a model structure. Compounds 5b and 5f were subsequently modeled, and their geometries optimized at the ωB97XD/6‐31+G(d,p) level of theory, again employing the C‐PCM “polar organic solvents” option (*∈ *= 37). These optimized geometries were then used as input structures for the molecular docking studies against HDAC6 and PI3Kα, selected based on the potency of **5b** and **5f** toward these targets.

### 6.8 Docking and MD Simulations

Molecular docking studies were performed using the FlarePro+ software (Cresset, Litlington, Cambridgeshire, UK) to predict the binding modes of the synthesized compounds within the target proteins. The crystallographic structures retrieved from the Protein Data Bank were HDAC6 (PDB ID: 5EDU) and PI3Kα (PDB ID: 4L23). Prior to docking, missing loops and gaps in the protein structures were reconstructed using the loop modeling tool implemented in FlarePro+, and the N‐ and C‐terminal residues were capped with acetyl (ACE) and N‐methylamide (NME) groups, respectively, to avoid artificial terminal charges. The binding sites were defined based on the positions of the co‐crystallized ligands, including all residues located within 6 Å of each reference ligand. The co‐crystallized ligands were also used as templates to guide the docking calculations. A redocking validation step was carried out to verify the reliability of the docking parameters. The accuracy of the protocol was evaluated by calculating the RMSD between the predicted and experimental poses, complemented by visual inspection of the resulting conformations. Docking runs were performed using the normal calculation method using default parameters and ligand flexibility settings implemented in FlarePro+. Since only one scoring function is available in the program, this function was applied consistently across all docking calculations. After validation, the compounds of interest were docked into the active sites of both targets, and key intermolecular distances were measured to characterize the predicted binding interactions. The resulting protein–ligand complexes were subsequently subjected to MD simulations using FlarePro+ with the OpenMM engine (v10.0.1). MD simulations were performed employing the Open Force Field 2.2.0 for the ligands and the AMBER force field for the proteins. The systems were solvated using the TIP3P explicit water model within a truncated octahedral box, and AM1‐BCC charges were assigned to the ligands. Following energy minimization, the systems underwent 200 ps of equilibration, followed by 100 ns of production simulation using a 4 fs integration time step under standard temperature and pressure conditions. Upon completion, the simulation trajectories were analyzed for RMSD and RMSF variations, as well as protein–ligand interaction profiles throughout the trajectories.

### 6.9 Cell Lines and Culture Conditions

JIMT‐1 (DSMZ ACC 589) and MDA‐MB‐231 (ATCC HTB‐26) breast cancer cell lines were maintained in Dulbecco's Modified Eagle Medium (DMEM) High Glucose with L‐glutamine (D5796, Sigma) supplemented with 10% fetal calf serum. MCF7‐derivative LY2 breast cancer cells were previously described (REF: PMID: 33420368). LY2 cells were cultured in phenol red‐free MEM (PRF‐MEM; 51 200‐046, Gibco) supplemented with 10% charcoal dextran‐stripped fetal calf serum (CDS‐FCS; F6765, Merck), 2 mmol/L L‐glutamine (G7513, Merck), and 10^−8^ mol/L 4‐hydroxytamoxifen (4‐OHT; H7904, Merck). All cell lines were maintained at 37°C in a humidified incubator with 5% CO_2_ and regularly authenticated and tested for mycoplasma.

#### 6.9.1 Cell Viability Assay

Cell viability was evaluated in response to increasing concentrations of 5a‐5f (0.3, 1, 3, 10, and 30 μM) or vehicle control (0.1% DMSO). LY2, JIMT1, and MDA‐MB‐231 cells were seeded at a density of 2,500 cells per well in 96‐well plates with a total volume of 100 μL per well. Prior to treatment, cells were serum‐deprived for 24 h. Following serum depletion, cells were treated in complete growth medium for 72 h. Cell viability was assessed using the MTS assay (G1111, Promega) according to the manufacturer's instructions. Absorbance was measured at 495 nm using a BioTek Synergy HTX Multi‐Mode Microplate Reader (Agilent). Results were normalized to the vehicle control (DMSO), and the half‐maximal inhibitory concentration (IC_50_) of the inhibitors was determined by nonlinear regression (curve fit) using a four‐parameter variable slope model.

### 6.10 Western Blotting Analysis

Total protein samples were extracted from treated cells using a lysis buffer containing 0.1% IGEPAL CA‐630 (I8896, Merck), 0.5% deoxycholic acid (D2510, Merck), and 0.1% SDS (L3771, Merck), freshly supplemented with protease and phosphatase inhibitor cocktail (78442, Thermo Fisher Scientific). Protein concentrations were quantified using the Pierce BCA Protein Assay Kit (23227, Thermo Fisher Scientific) according to the manufacturer's instructions. Equal amounts of protein (30 µg per sample) were separated by SDS‐PAGE using Bolt Bis‐Tris Plus Mini 4–12% gradient gels (NW04125BOX, Invitrogen) and transferred onto nitrocellulose membranes using the iBlot 3 Western Blot Transfer System (Thermo Fisher Scientific). Subsequently, membranes were blocked with either 5% bovine serum albumin (BSA; A2153, Sigma‐Aldrich) or skim milk powder (LP0033B, Thermo Fisher Scientific), depending on the antibody requirements, in Tris‐buffered saline with 0.1% Tween‐20 (TBS‐T) for 1 h at room temperature. Blocked membranes were then incubated overnight at 4°C with primary antibodies diluted in the corresponding blocking buffer. The following primary antibodies were used: rabbit anti‐phospho‐Akt (1:1000; #9271, Cell Signaling), mouse anti‐Akt (1:1000; #2920, Cell Signaling), rabbit anti‐acetyl α‐Tubulin (1:1000; #5335, Cell Signaling), rabbit anti‐acetyl histone H3 (Lys9) (1:1000; #9649, Cell Signaling), rabbit anti‐acetyl histone H3 (Lys14) (1:1000; #7627, Cell Signaling), and mouse anti‐GAPDH (1:1000; #MAB374, EMD Millipore), which served as a loading control. Membranes were washed three times with TBS‐T (10 min each) and then incubated with the appropriate horseradish peroxidase (HRP)–conjugated secondary antibody for 1 h at room temperature. The following secondary antibodies were used: HRP‐conjugated anti‐rabbit IgG (1:2000; #NA934, Cytiva) and HRP‐conjugated anti‐mouse IgG (1:2000; #NXA931, Cytiva). After incubation with secondary antibodies, membranes were washed three times with TBS‐T (10 min each) and developed using the Pierce ECL Western Blotting Substrate (#32106, Thermo Fisher Scientific). Chemiluminescence signals were detected using the Amersham Imager 680 Imaging System. Densitometric analysis of protein bands was conducted using ImageJ software, and protein expression levels were normalized to the corresponding loading controls. All experiments were conducted in triplicate (N = 3).

#### 6.10.1 Statistical Analysis

One‐way analysis of variance (ANOVA), nonparametric test, multiple comparison (comparing the mean rank of each column with the mean rank of a control column 0).

## Supporting Information

Additional supporting information can be found online in the Supporting Information section.

## Conflicts of Interest

The authors declare no conflicts of interest.

## Supporting information

Supplementary Material

## Data Availability

The data that support the findings of this study are available in the supplementary material of this article.
