# Supplementary material for: Design, Synthesis, and Biological Evaluation of Novel Triazine‐Based Dual Histone Deacetylase/phosphatidylinositol 3‐kinase Inhibitors for Breast Cancer Therapy
Source: ChemMedChem. 2026 Apr 25;21(8):e202501041. doi: 10.1002/cmdc.202501041 (PMC13110364; doi:10.1002/cmdc.202501041)
Supplement: Supplementary file 1 — Supplementary Material [file CMDC-21-e202501041-s001.pdf]

Design, synthesis, and biological evaluation of novel triazine-based dual HDAC/PI3K inhibitors for breast cancer therapy

Lara Luzietti[1,‡], Gustavo Salgado Pires[2,3,‡], Ana Ryan[1], Crisamie Regidor[1], Matthew Hiller[4], Danilo Sarti[1], Tríona Ní Chonghaile[4], Pedro de Sena Murteira Pinheiro[2,3], Damir Varešlija[1,*], Daniel Alencar Rodrigues[1,*]

1. School of Pharmacy and Biomolecular Sciences (PBS), Royal College of Surgeons in Ireland, 123 St Stephen's Green, Dublin 2, Ireland.
2. Laboratório de Avaliação e Síntese de Substâncias Bioativas (LASSBio), Instituto de Ciências Biomédicas, Universidade Federal do Rio de Janeiro, Cidade Universitária, Rio de Janeiro 21941-902, Brazil
3. Programa de Pós-Graduação em Farmacologia e Química Medicinal (PPGFQM), Instituto de Ciências Biomédicas, Universidade Federal do Rio de Janeiro, Cidade Universitária, Rio de Janeiro 21941-902, Brazil
4. Department of Physiology and Medical Physics, Royal College of Surgeons in Ireland, 123 St Stephen's Green, Dublin 2, Ireland.

‡These authors contributed equally to this study.

*To whom correspondence should be addressed. E-mail: damirvareslija@rcsi.com; danielalencar@rcsi.com.

Contents

**Supplementary Figures**	**3**
**^{1}H and ^{13}C NMR spectral data**	**8**
**High-Resolution Mass Spectrometry**	**21**
**Purity by High-Performance Liquid Chromatography**	**28**

Supplementary Figures and Tables

DRL-01 - 5a

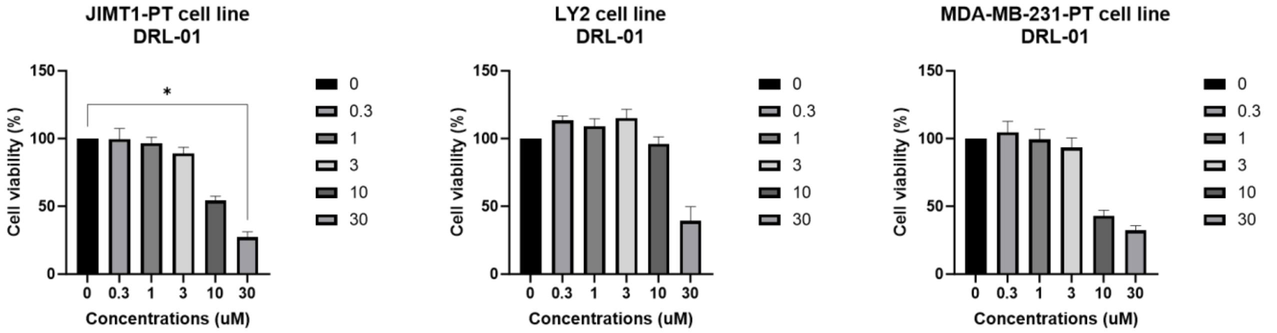

DRL-02 – 5b

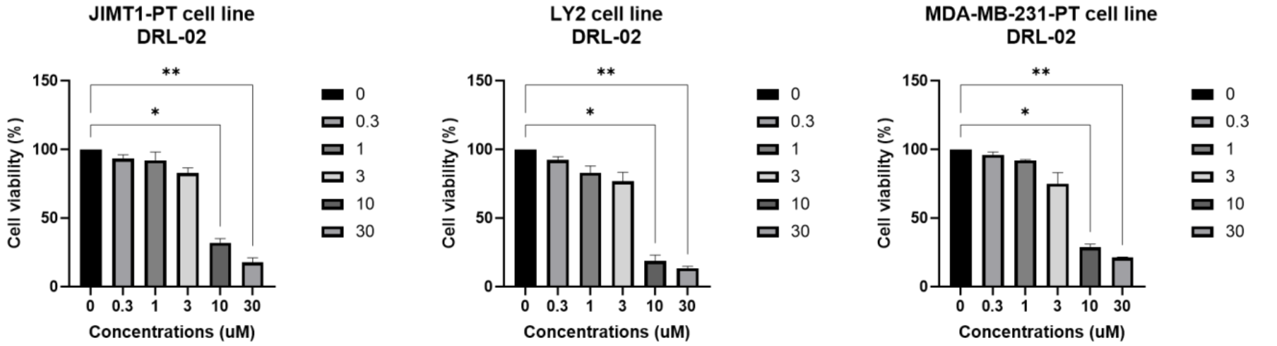

DRL-03 – 5c

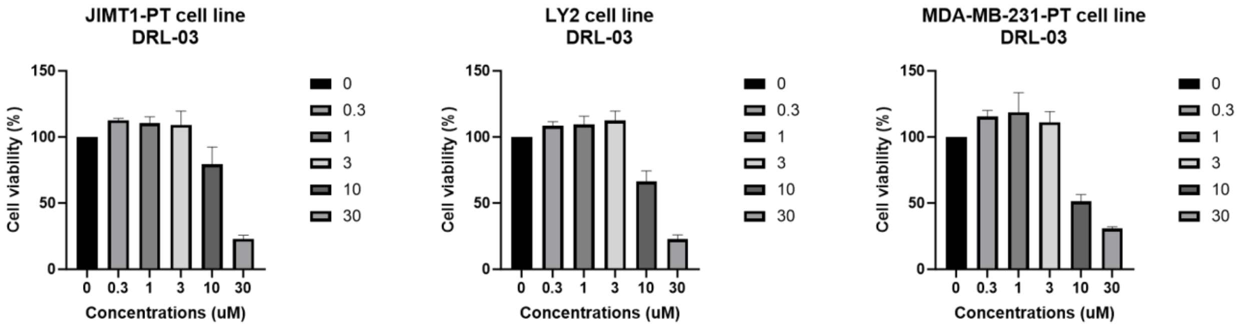

DRL-04 – 5d

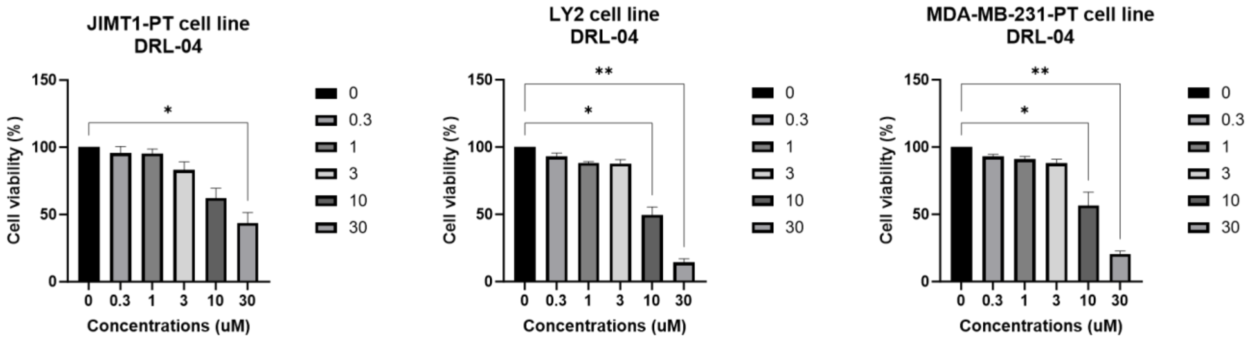

DRL-05 – 5e

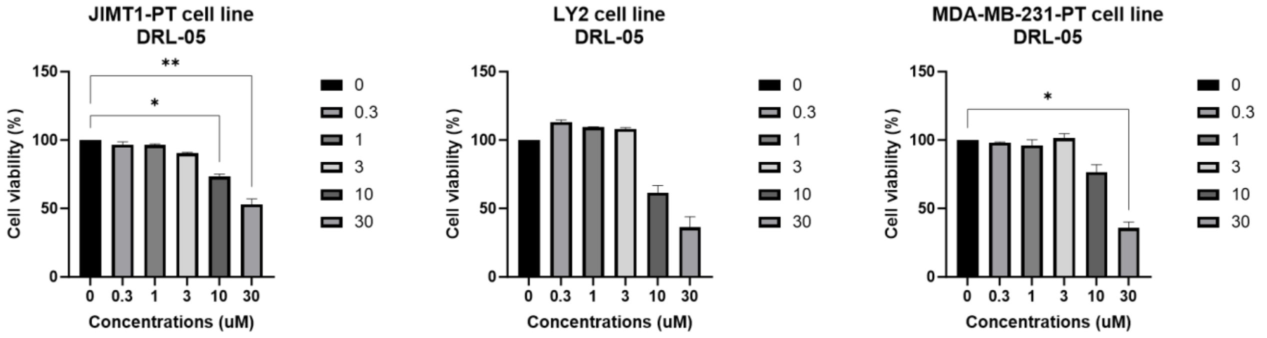

DRL-06 – 5f

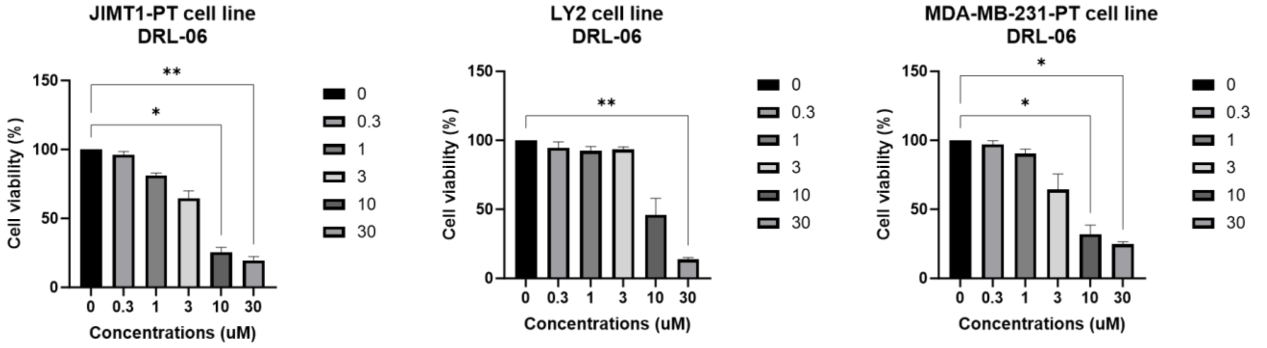

Fimepinostat

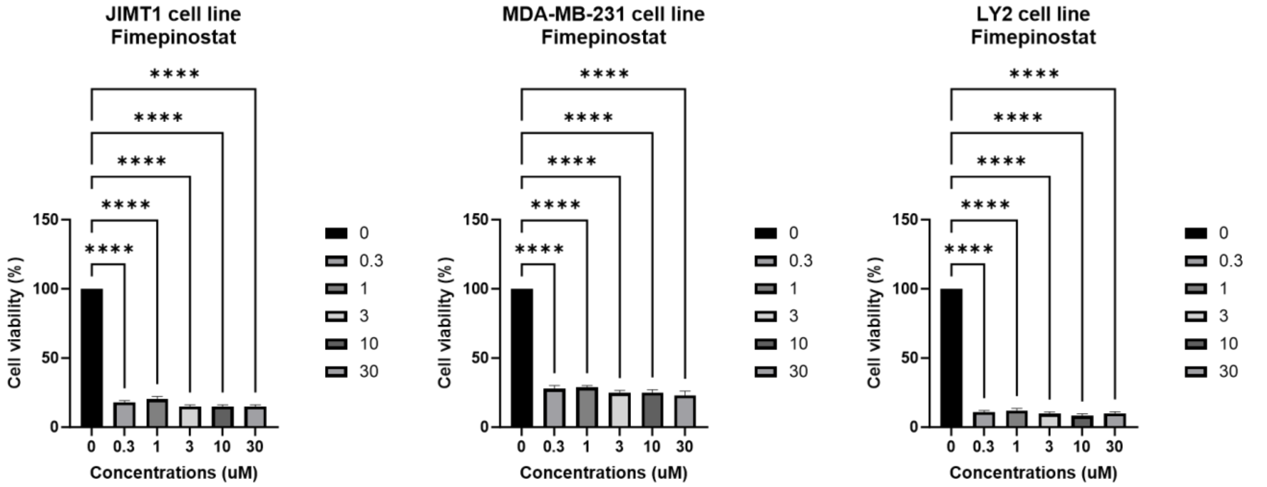

Figure S1. Dose-dependent effects of compound treatment (5a-5f) on breast cancer cell viability across molecular subtypes. Bar graphs depict the relative viability of three breast cancer cell lines following treatment with increasing concentrations of the compound (0, 0.3, 1, 3, 10, and 30 μM). JJIMT1-PT ($HER2^+$ subtype), MDA-MB-231-PT (TNBC subtype), and LY2 (ER^+ subtype) represent distinct molecular subtypes.

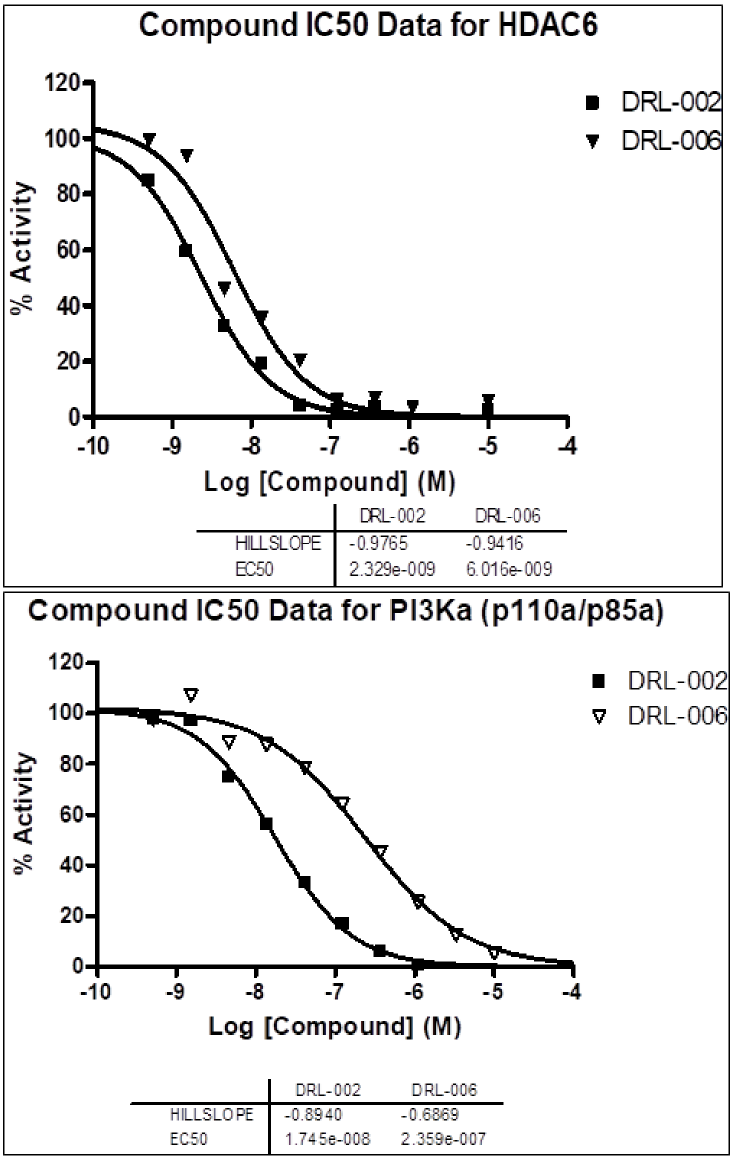

Figure S2. IC_{50} curves for **5b** (DRL-002) and **5f** (DRL-006) in HDAC6 and PI3Kα. Compounds 5b and 5f were tested in a 10-dose IC_{50} format using threefold serial dilutions starting at 1 μM (singlet).

Table SX. Relative stability of **5a**-derived tautomers calculated at the ωB97XD/6-31+G(d,p) level of theory in polar solvent. Calculations were performed in Spartan’24.

**Tautomer**	**2D Structure**	**ΔE (kcal/mol)**	**Boltzmann Weights**
5a – I	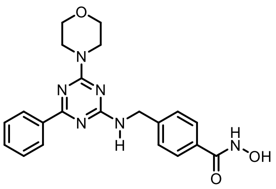	0	100
5a – II	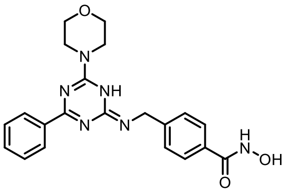	16.86	0
5a – III	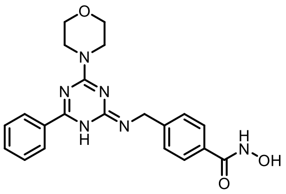	17.23	0
5a – IV	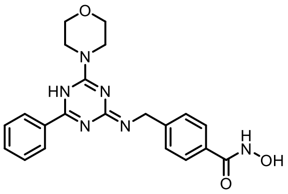	22.40	0
5a – V	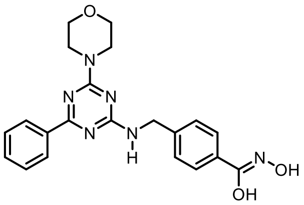	9.73	0
5a – VI	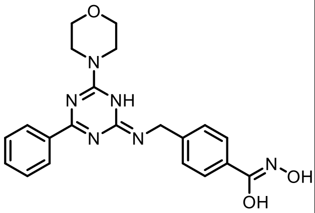	26.68	0
5a – VII	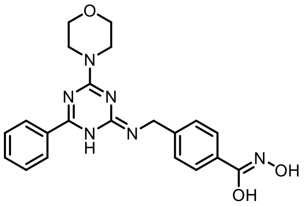	26.95	0

5a - VIII	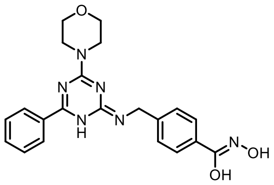	32.15	0

^{1}H and ^{13}C NMR spectral data

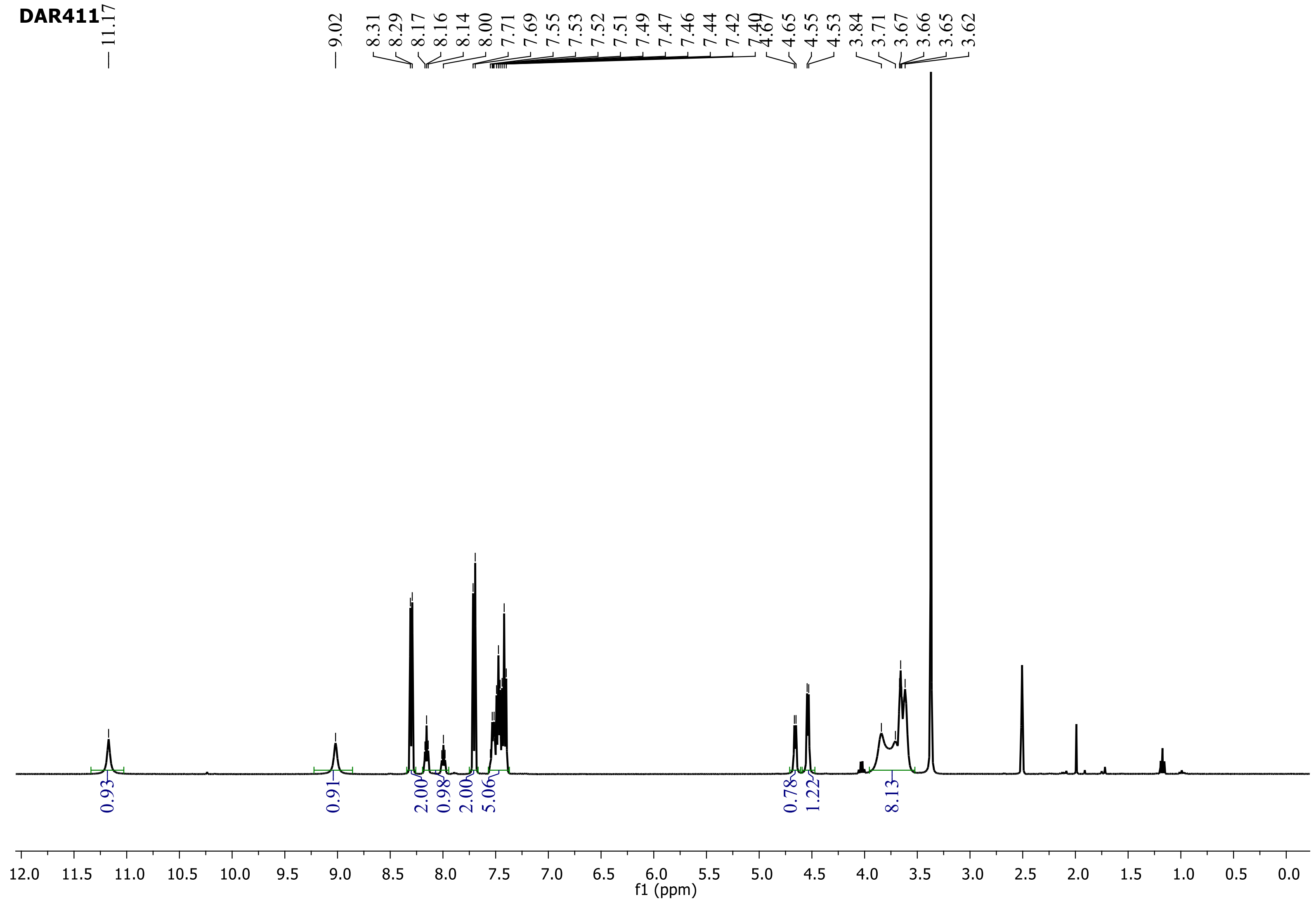

Spectrum 1 - ^{1}H NMR (400MHz, DMSO-d_6) of *N*-hydroxy-4-(((4-morpholino-6-phenyl-1,3,5-triazin-2-yl)amino)methyl)benzamide (DRL-01) (**5a**).

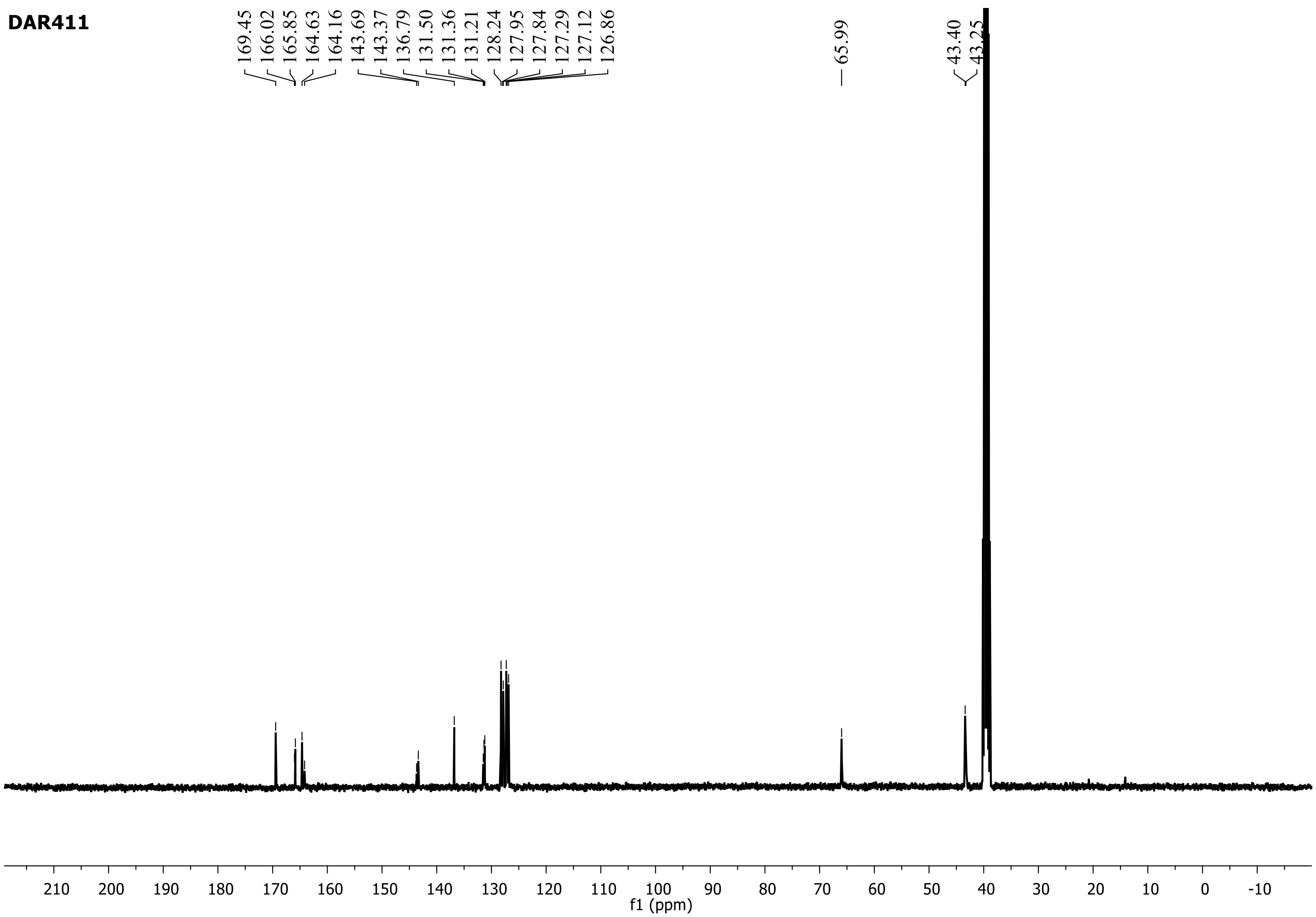

Spectrum 2 – ^{13}C NMR (100MHz, DMSO-d_6) of *N*-hydroxy-4-(((4-morpholino-6-phenyl-1,3,5-triazin-2-yl)amino)methyl)benzamide (DRL-01) (**5a**).

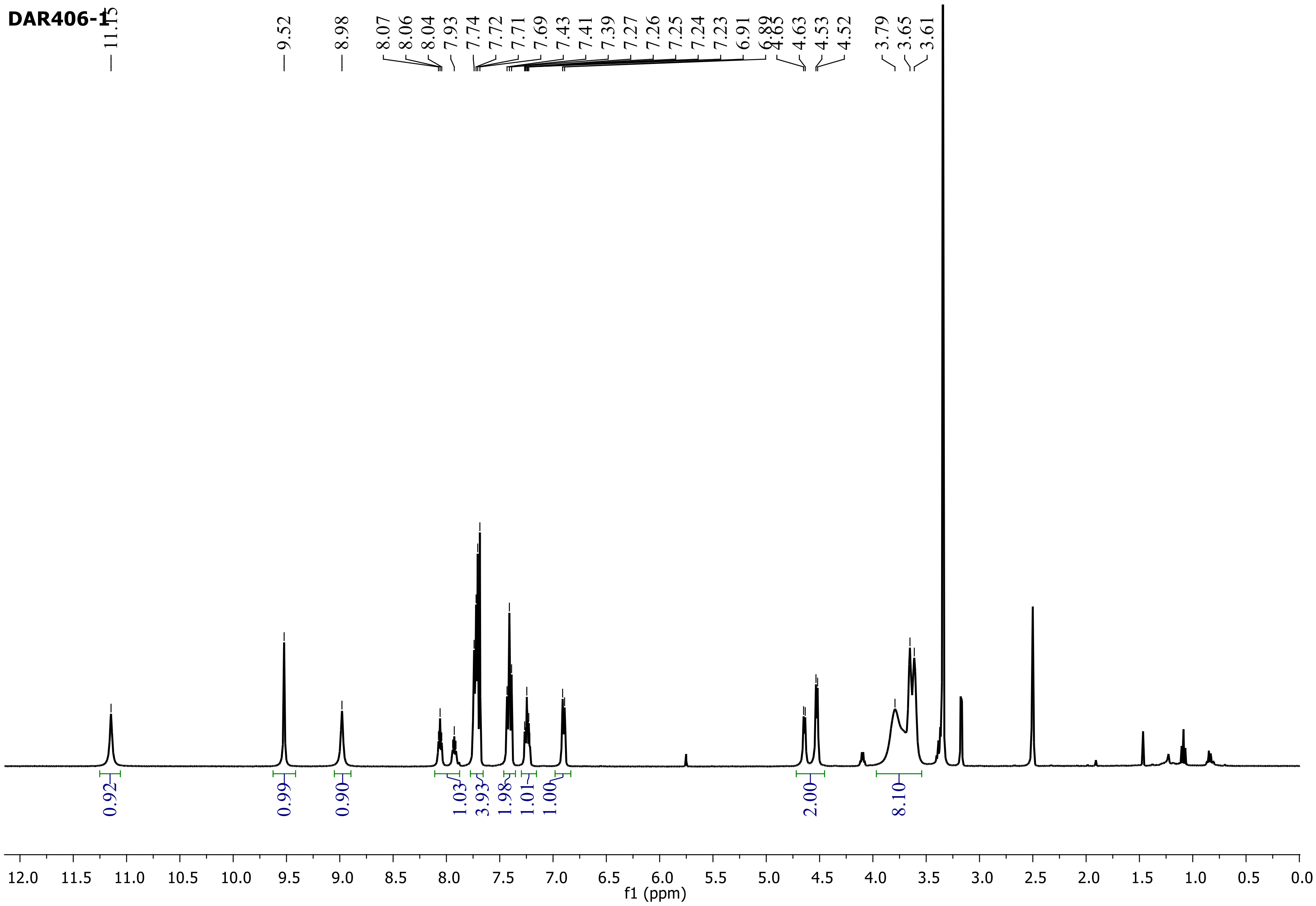

Spectrum 3 - ^{1}H NMR (400MHz, DMSO-d_6) of *N*-hydroxy-4-(((4-(3-hydroxyphenyl)-6-morpholino-1,3,5-triazin-2-yl)amino)methyl)benzamide (DRL-02) (**5b**).

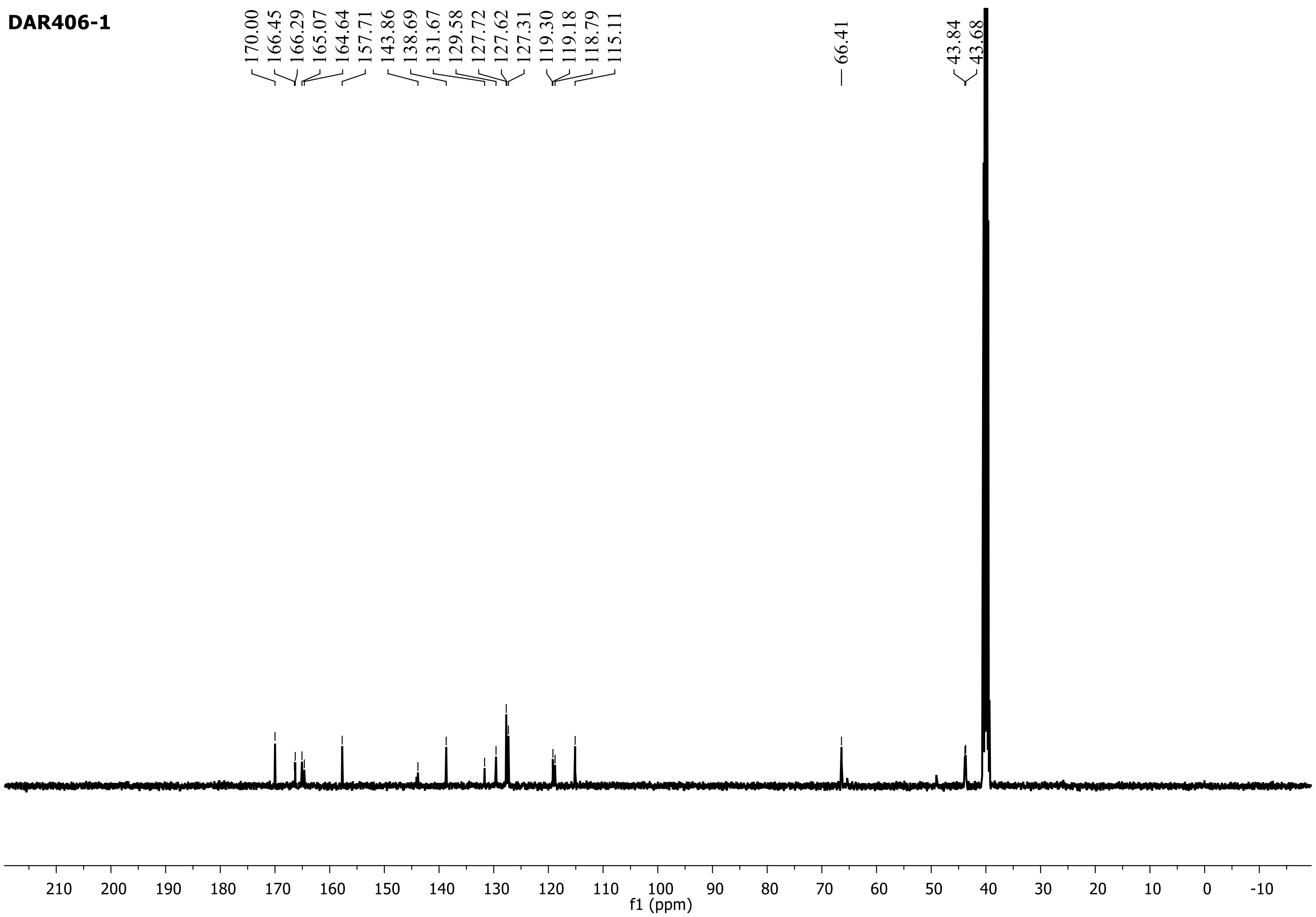

Spectrum 4 – ^{13}C NMR (100MHz, DMSO-d_6) of *N*-hydroxy-4-(((4-(3-hydroxyphenyl)-6-morpholino-1,3,5-triazin-2-yl)amino)methyl)benzamide (DRL-02) (**5b**).

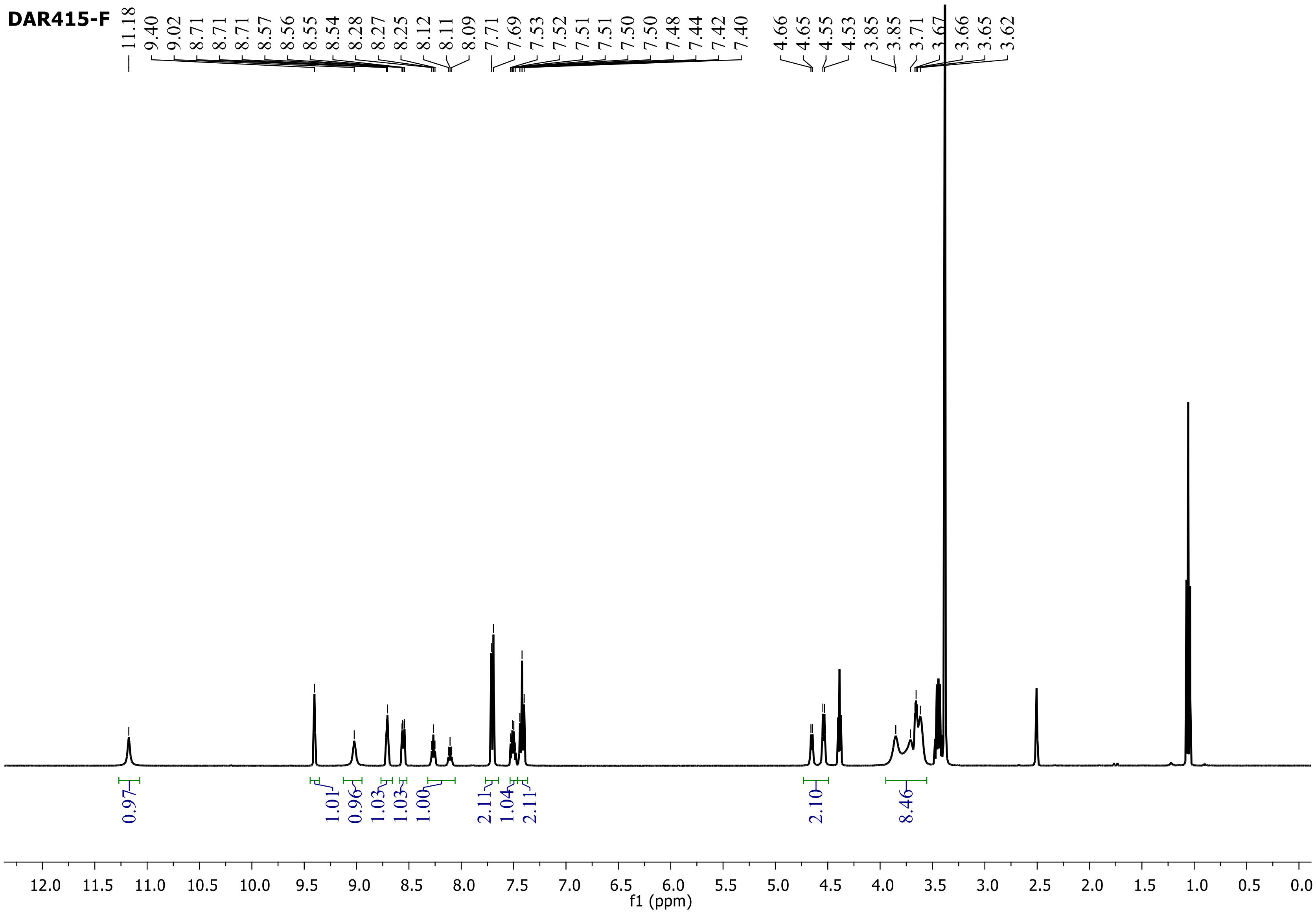

Spectrum 5 - ^{1}H NMR (400MHz, DMSO-d_6) of *N*-hydroxy-4-(((4-morpholino-6-(pyridin-3-yl)-1,3,5-triazin-2-yl)amino)methyl)benzamide (DRL-03) (**5c**).

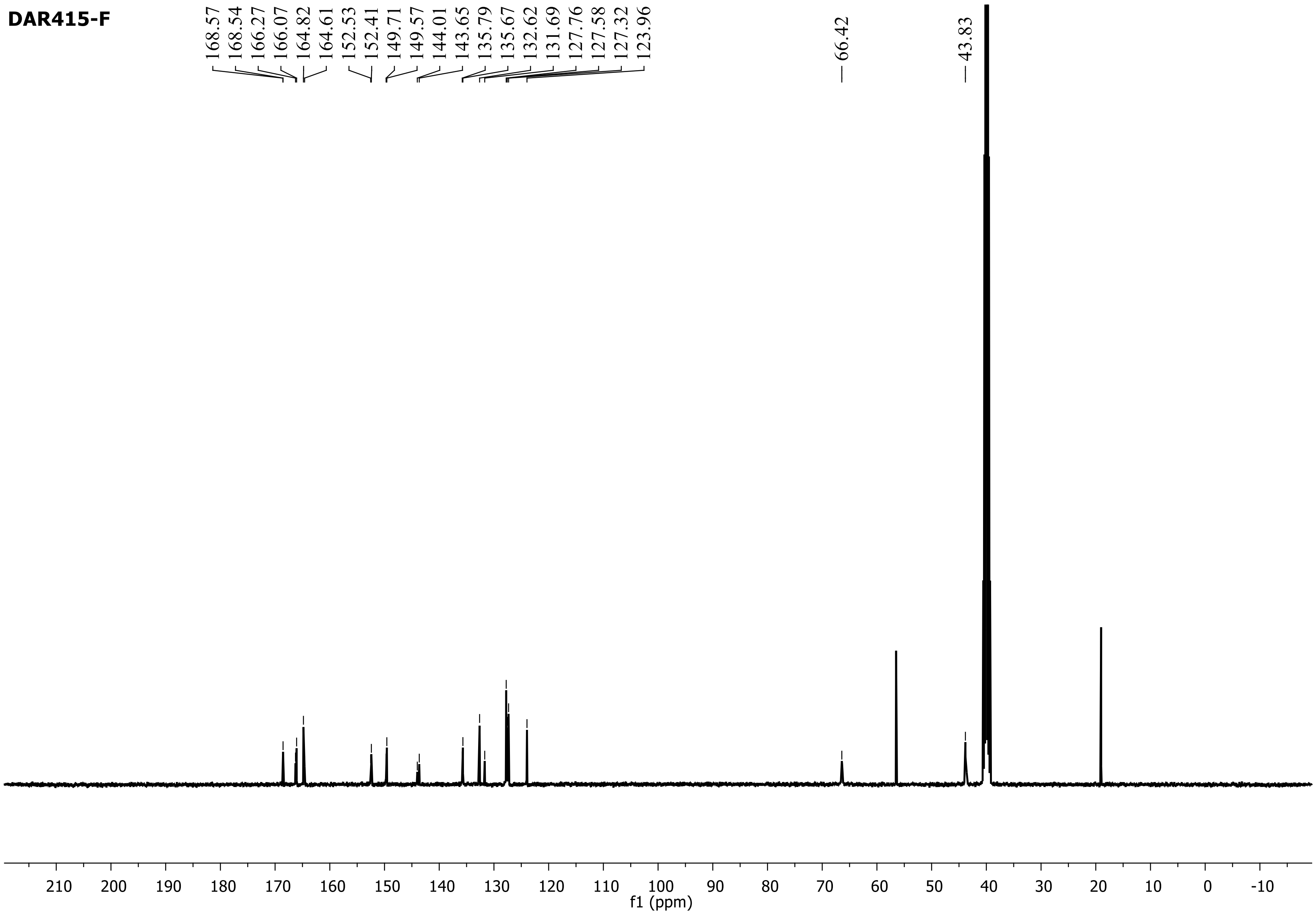

Spectrum 6 – ^{13}C NMR (100MHz, DMSO-d_6) of *N*-hydroxy-4-(((4-morpholino-6-(pyridin-3-yl)-1,3,5-triazin-2-yl)amino)methyl)benzamide (DRL-03) (**5c**).

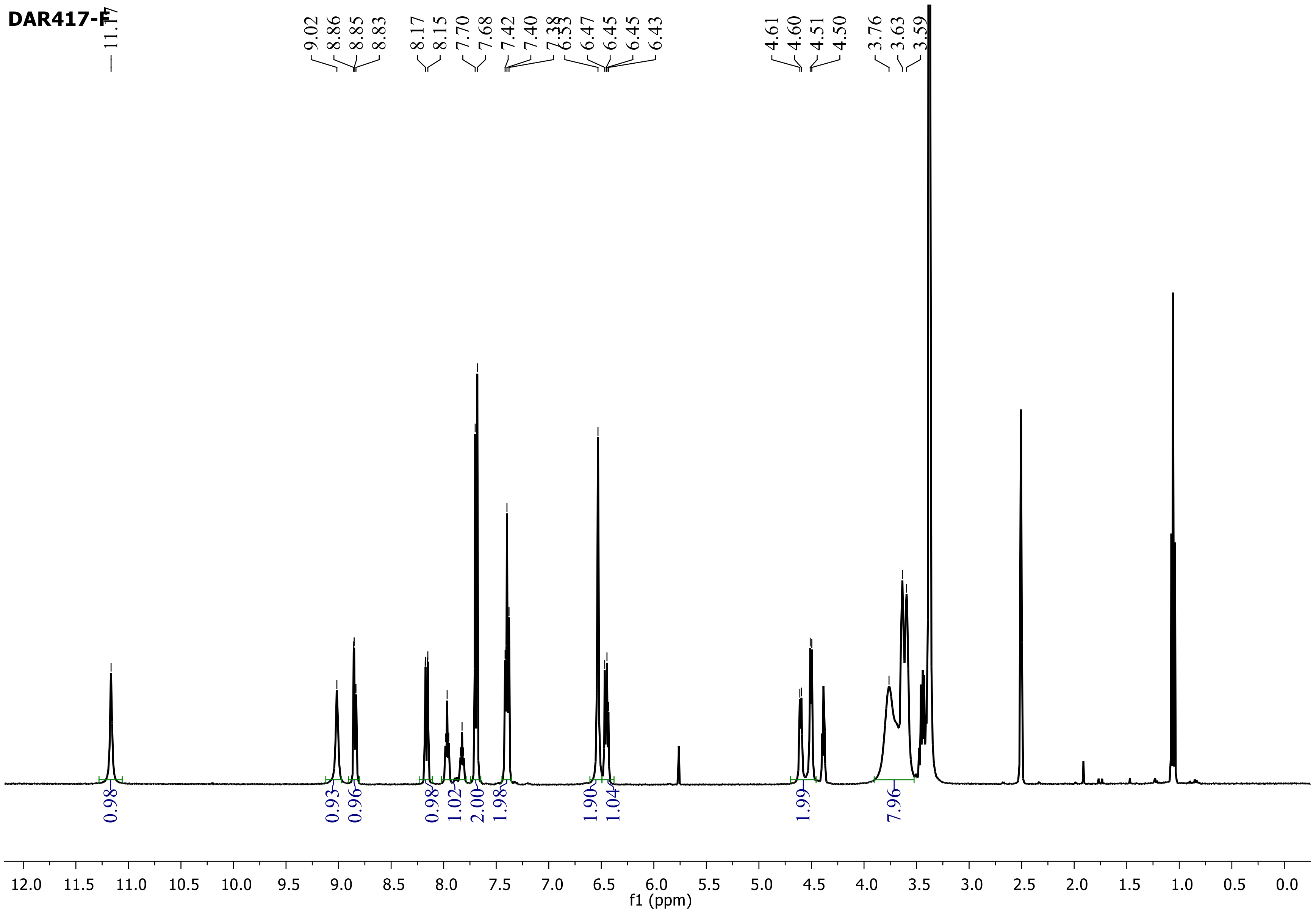

Spectrum 7 - ^{1}H NMR (400MHz, DMSO-d_6) of 4-(((4-(6-aminopyridin-3-yl)-6-morpholino-1,3,5-triazin-2-yl)amino)methyl)-*N*-hydroxybenzamide (DRL-04) (**5d**).

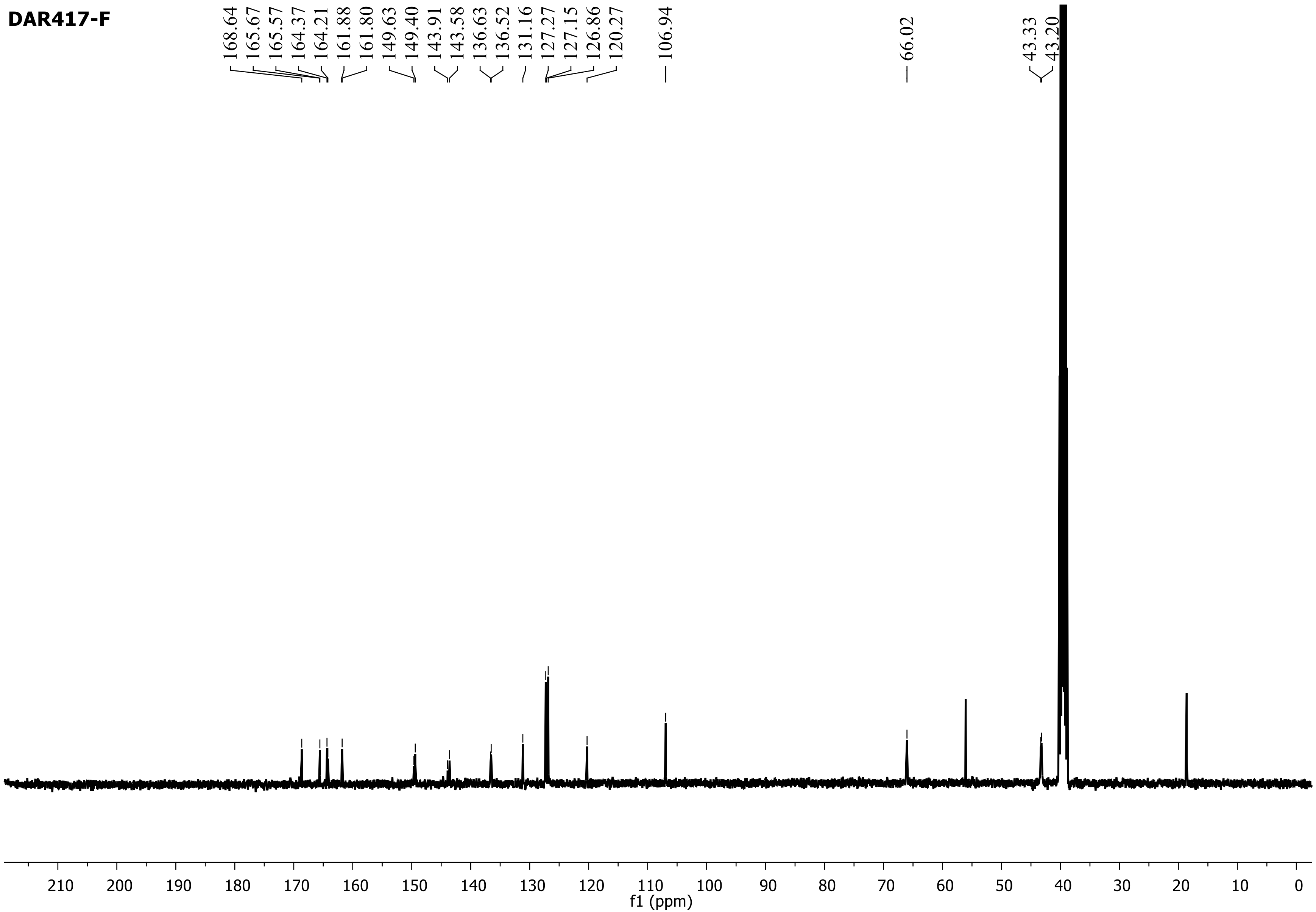

Spectrum 8 – ^{13}C NMR (100MHz, DMSO-d_6) of 4-(((4-(6-aminopyridin-3-yl)-6-morpholino-1,3,5-triazin-2-yl)amino)methyl)-*N*-hydroxybenzamide (DRL-04) (**5d**).

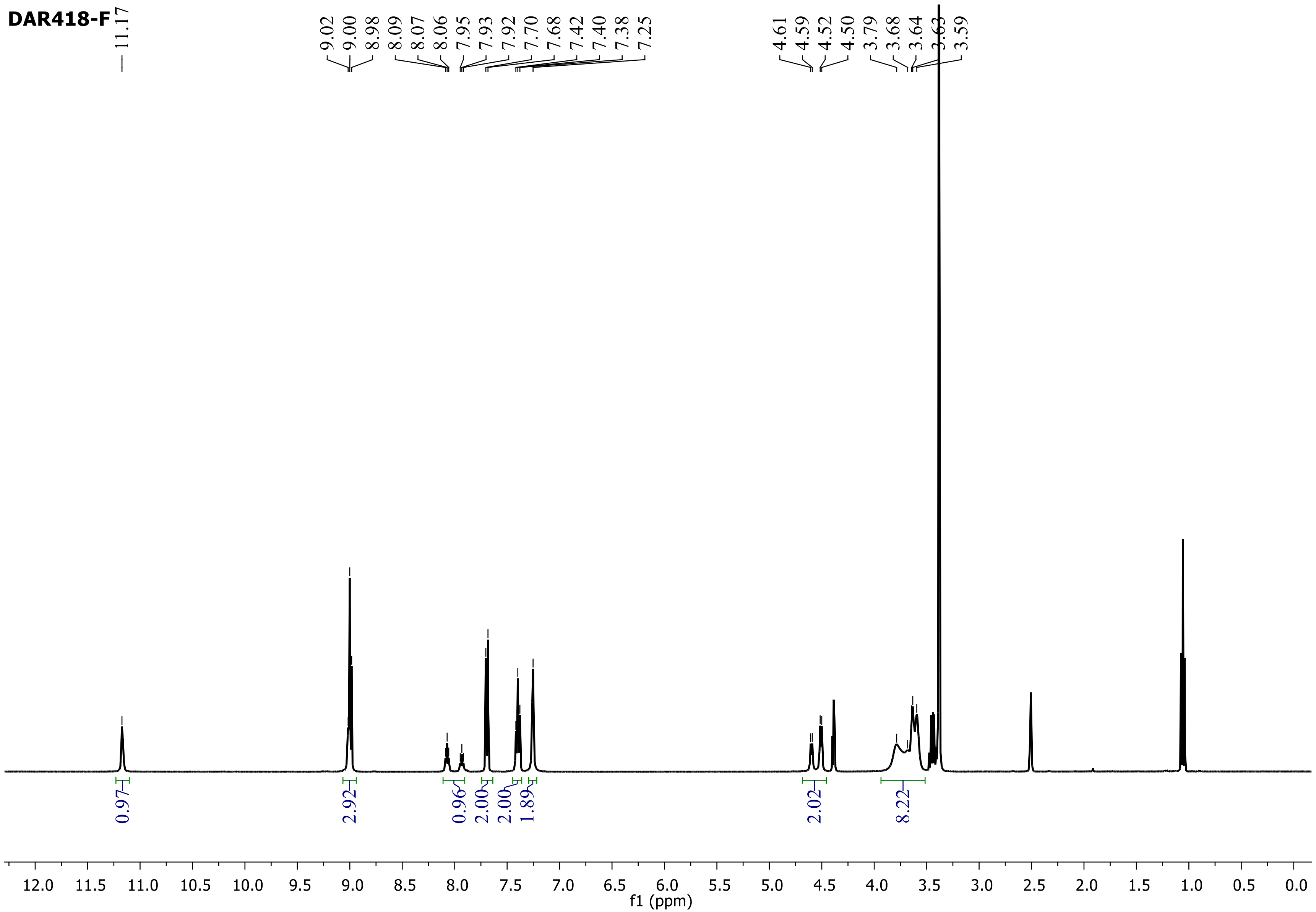

Spectrum 9 - ^{1}H NMR (400MHz, DMSO-d_6) of 4-(((4-(2-aminopyrimidin-5-yl)-6-morpholino-1,3,5-triazin-2-yl)amino)methyl)-*N*-hydroxybenzamide (DRL-05) (**5e**).

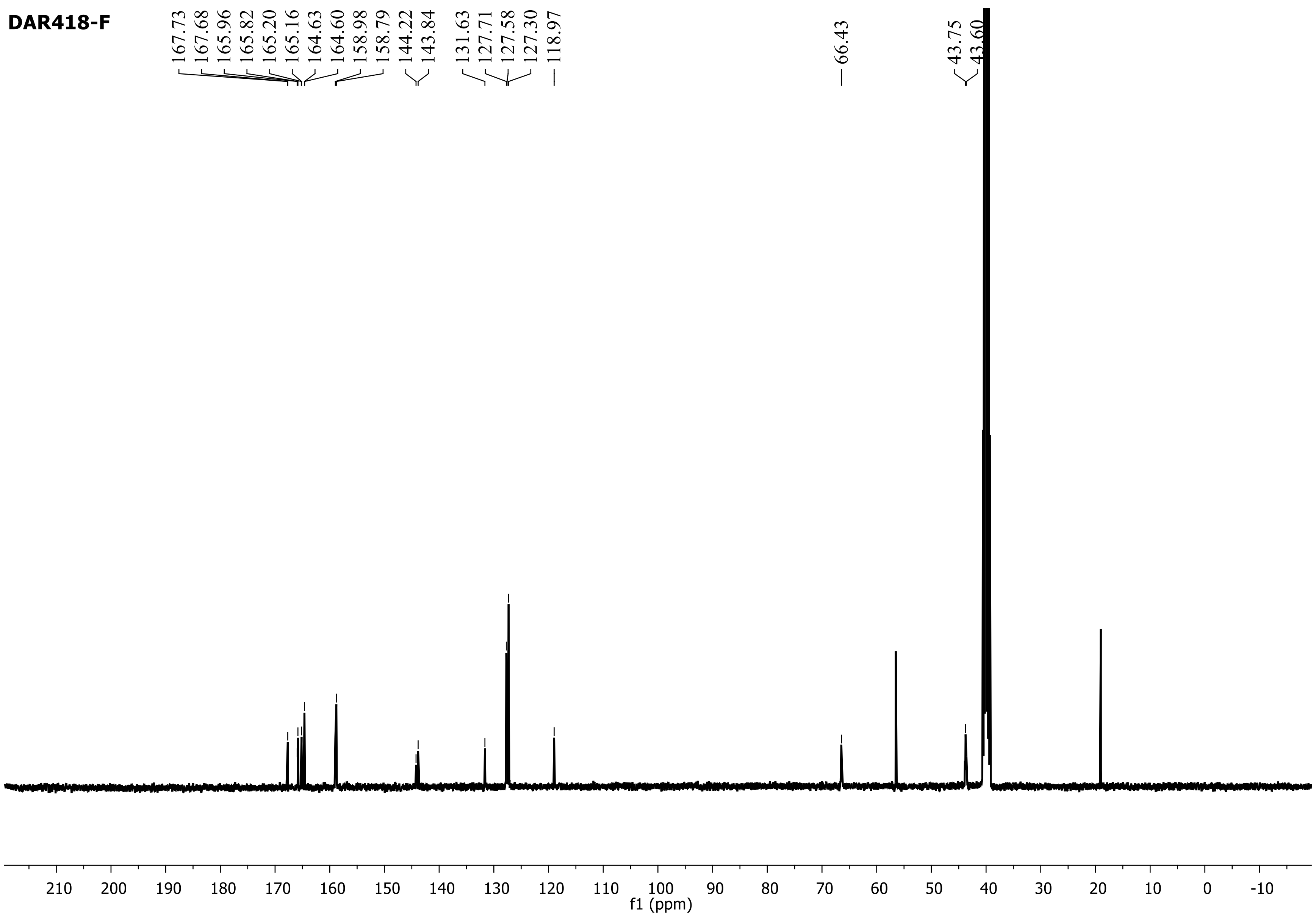

Spectrum 10 – ^{13}C NMR (100MHz, DMSO-d_6) of 4-(((4-(2-aminopyrimidin-5-yl)-6-morpholino-1,3,5-triazin-2-yl)amino)methyl)-*N*-hydroxybenzamide (DRL-05) (**5e**).

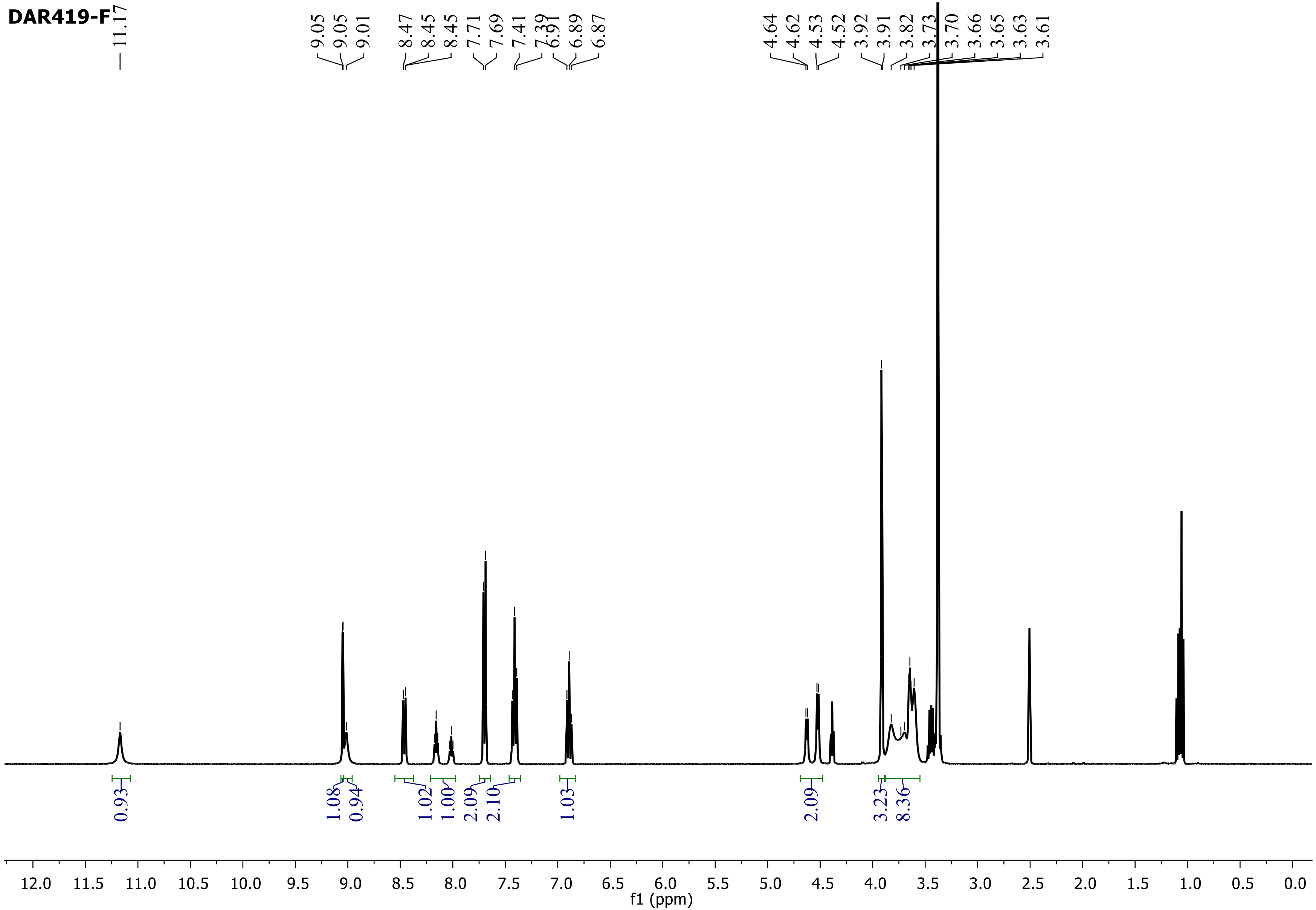

Spectrum 11 - ^{1}H NMR (400MHz, DMSO-d_6) of *N*-hydroxy-4-(((4-(6-methoxypyridin-3-yl)-6-morpholino-1,3,5-triazin-2-yl)amino)methyl)benzamide (DRL-06) (**5f**).

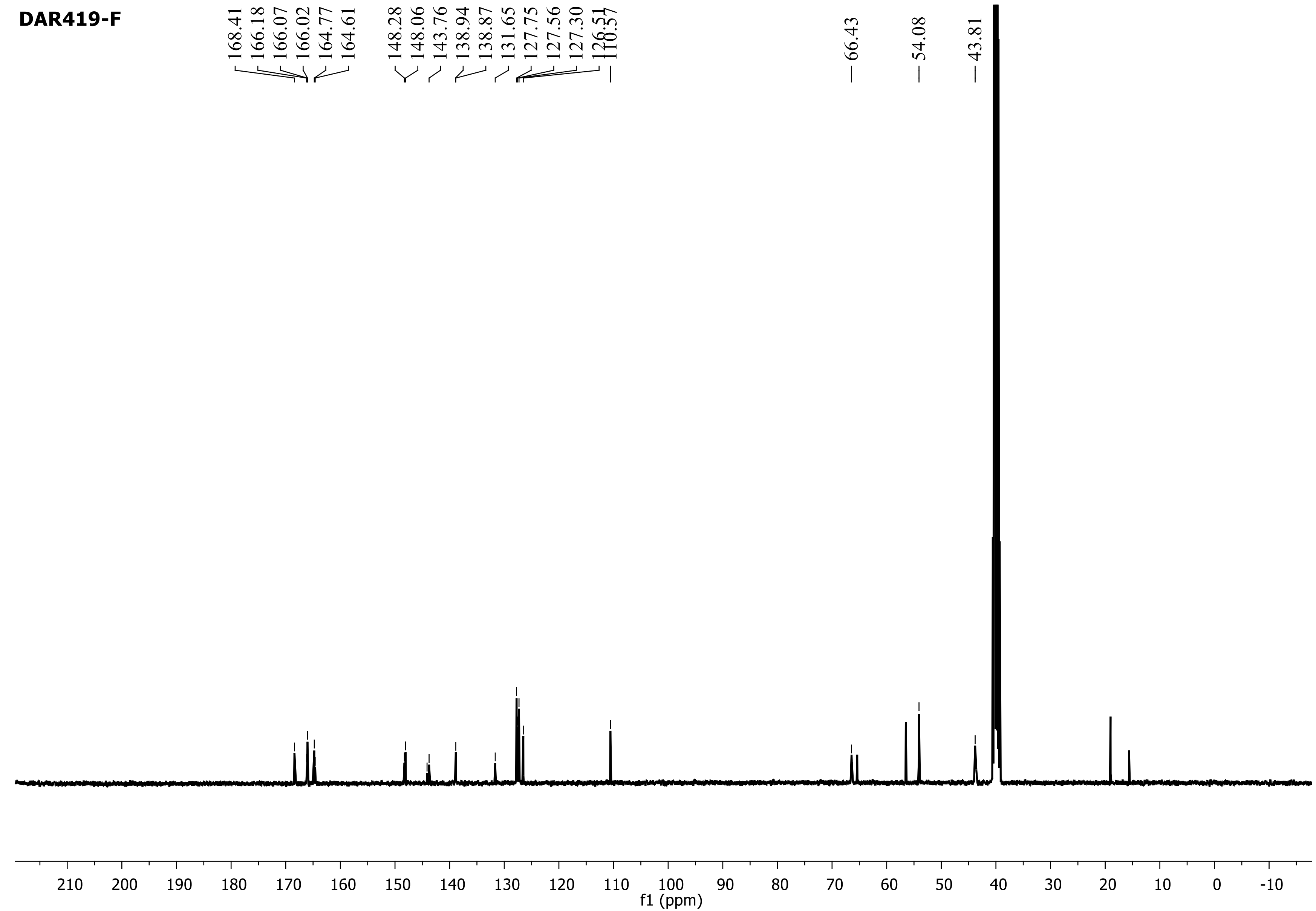

Spectrum 12 – ^{13}C NMR (100MHz, DMSO-d_6) of *N*-hydroxy-4-(((4-(6-methoxypyridin-3-yl)-6-morpholino-1,3,5-triazin-2-yl)amino)methyl)benzamide (DRL-06) (**5f**).

High-Resolution Mass Spectrometry

Trinity College Dublin

School of Chemistry Mass Spectrometry Unit

Sample-ID		Station	
Submitter		Supervisor	
Analysis Name	DRL-001_GA5_01_42999.d	Acquisition Date	02/07/2025 17:09:59
Sample Description			

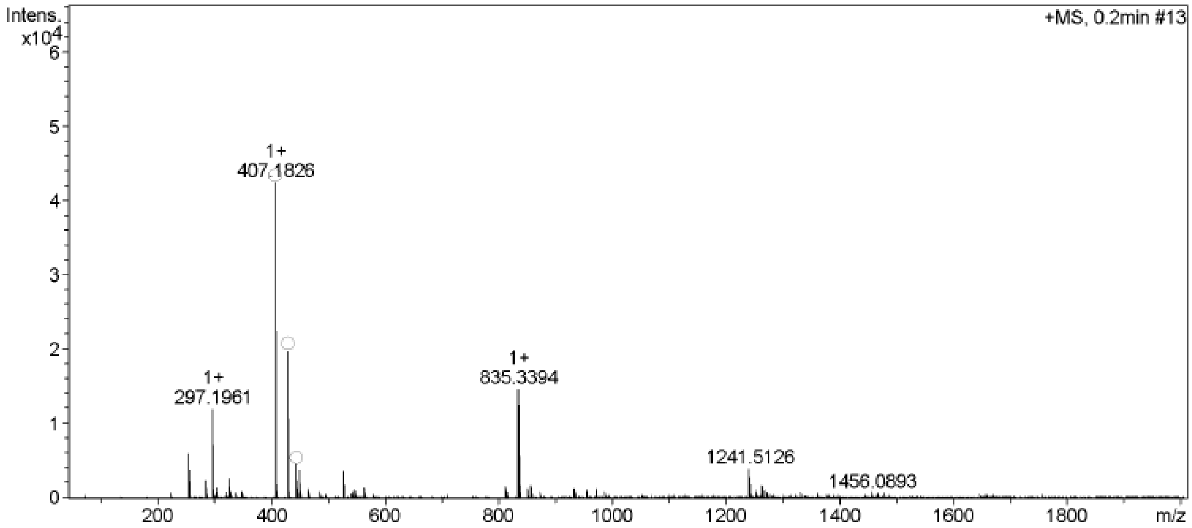

Meas. m/z	#	Ion Formula	m/z	err [mDa]	err [ppm]	rdb	N-Rule	e⁻ Conf	mSigma
407.182598	1	C21H23N6O3	407.182615	0.0	0.0	13.5	ok	even	4.8
429.164526	1	C21H22N6NaO3	429.164559	-0.0	-0.1	13.5	ok	even	11.5
445.137282	1	C21H22KN6O3	445.138497	1.2	2.7	13.5	ok	even	14.0

SmartFormula Settings

Low value of mSigma indicates good isotopic pattern match

Spectrum 13 - HRMS ESI of *N*-hydroxy-4-(((4-morpholino-6-phenyl-1,3,5-triazin-2-yl)amino)methyl)benzamide (DRL-01) (**5a**).

Trinity College Dublin

School of Chemistry Mass Spectrometry Unit

Sample-ID		Station	
Submitter		Supervisor	
Analysis Name	DRL-002_GA6_01_43000.d	Acquisition Date	02/07/2025 17:13:15
Sample Description			

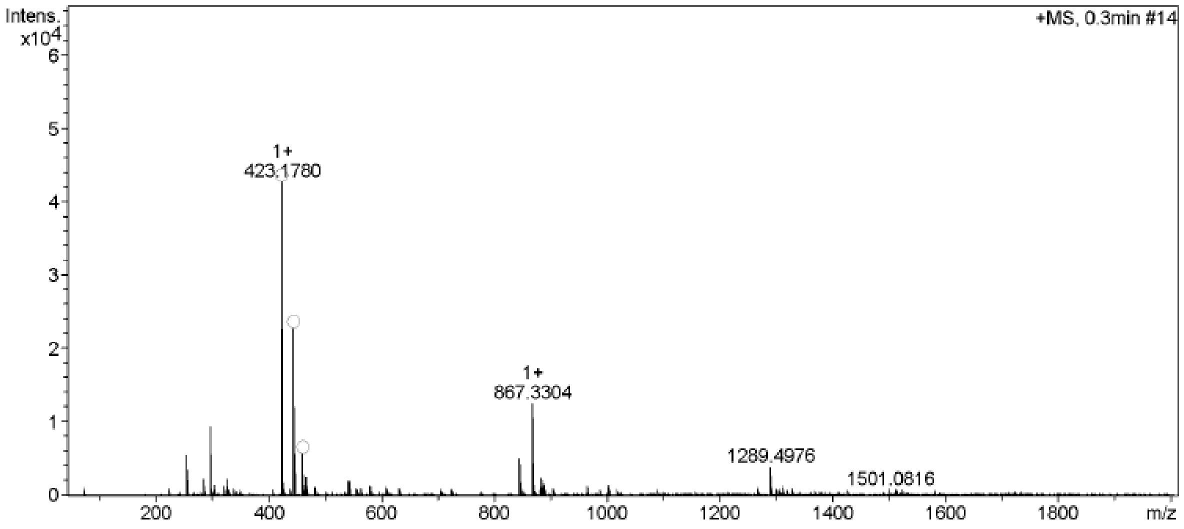

Meas. m/z	#	Ion Formula	m/z	err [mDa]	err [ppm]	rdb	N-Rule	e¯ Conf	mSigma
423.177985	1	C21H23N6O4	423.177530	0.5	1.1	13.5	ok	even	5.8
445.159582	1	C21H22N6NaO4	445.159474	0.1	0.2	13.5	ok	even	1.3
461.133033	1	C21H22KN6O4	461.133411	-0.4	-0.8	13.5	ok	even	11.3

SmartFormula Settings

Low value of mSigma indicates good isotopic pattern match

Spectrum 14 - HRMS ESI of *N*-hydroxy-4-(((4-(3-hydroxyphenyl)-6-morpholino-1,3,5-triazin-2-yl)amino)methyl)benzamide (DRL-02) (**5b**).

Trinity College Dublin

School of Chemistry Mass Spectrometry Unit

Sample-ID
Submitter
Analysis Name DRL-003_GA7_01_43001.d
Sample Description

Station
Supervisor
Acquisition Date 02/07/2025 17:16:32

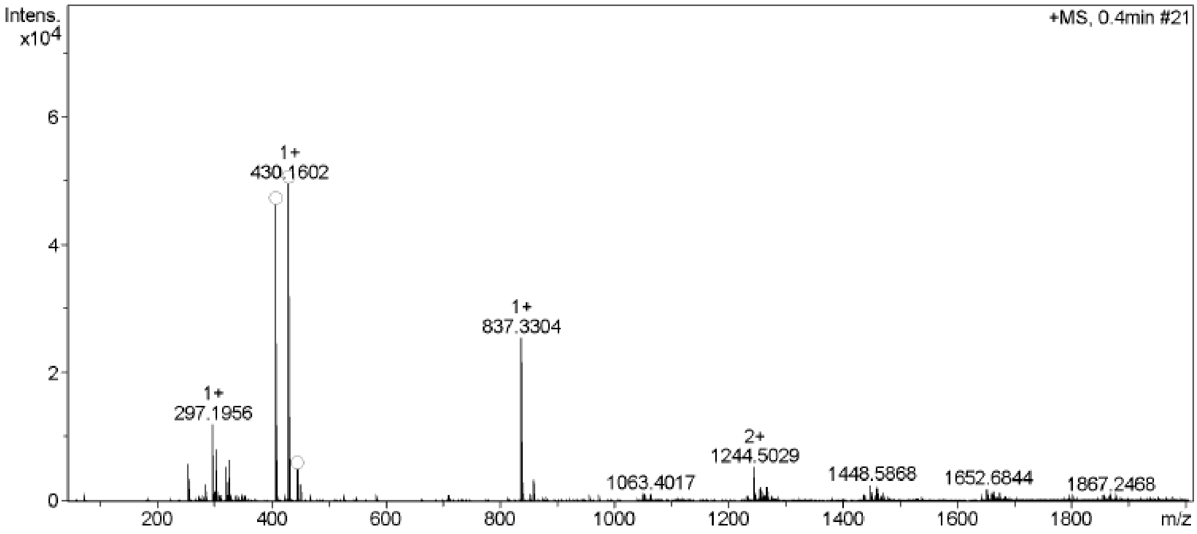

Meas. m/z	#	Ion Formula	m/z	err [mDa]	err [ppm]	rdb	N-Rule	e⁻ Conf	mSigma
408.178330	1	C20H22N7O3	408.177864	0.5	1.1	13.5	ok	even	8.9
430.160247	1	C20H21N7NaO3	430.159808	-0.4	-1.0	13.5	ok	even	2.7
446.133021	1	C20H21KN7O3	446.133746	0.7	1.6	13.5	ok	even	11.2

SmartFormula Settings

Low value of mSigma indicates good isotopic pattern match

Spectrum 15 - HRMS ESI of *N*-hydroxy-4-(((4-morpholino-6-(pyridin-3-yl)-1,3,5-triazin-2-yl)amino)methyl)benzamide (DRL-03) (**5c**).

Sample-ID		Station	
Submitter		Supervisor	
Analysis Name	DRL-004_RA6_01_43319.d	Acquisition Date	24/07/2025 12:08:06
Sample Description			

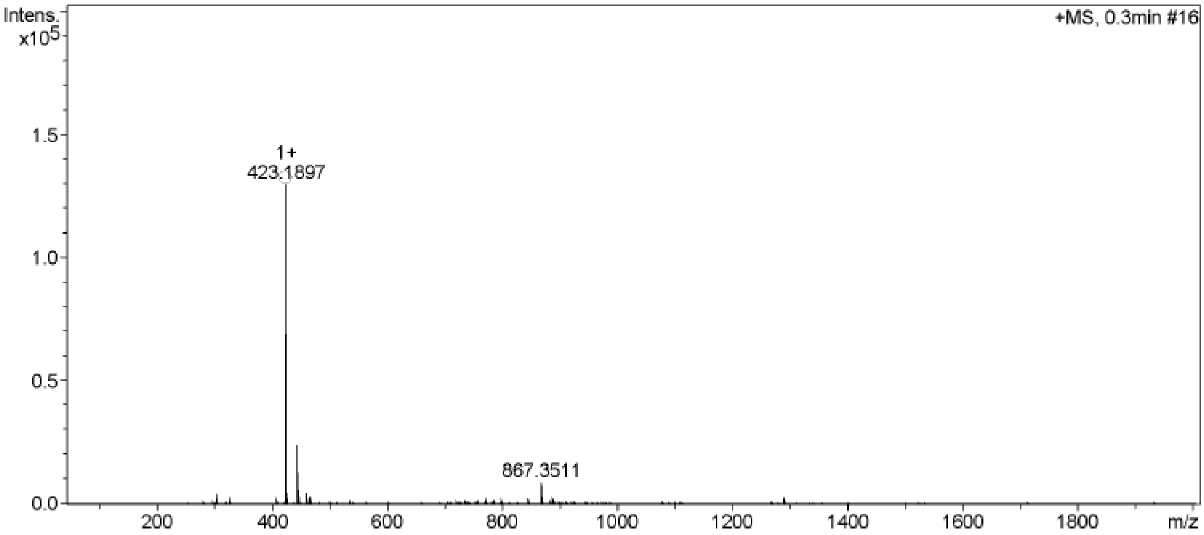

Meas. m/z	#	Ion Formula	m/z	err [mDa]	err [ppm]	rdb	N-Rule	e⁻ Conf	mSigma
423.189678	1	C20H23N8O3	423.188763	-0.9	-2.2	13.5	ok	even	4.7

SmartFormula Settings

Low value of mSigma indicates good isotopic pattern match

Spectrum 16 - HRMS ESI of 4-(((4-(6-aminopyridin-3-yl)-6-morpholino-1,3,5-triazin-2-yl)amino)methyl)-*N*-hydroxybenzamide (DRL-04) (**5d**).

Trinity College Dublin

School of Chemistry Mass Spectrometry Unit

Sample-ID

Submitter

Analysis Name DRL-005_RA7_01_43320.d

Sample Description

Station

Supervisor

Acquisition Date 24/07/2025 12:11:22

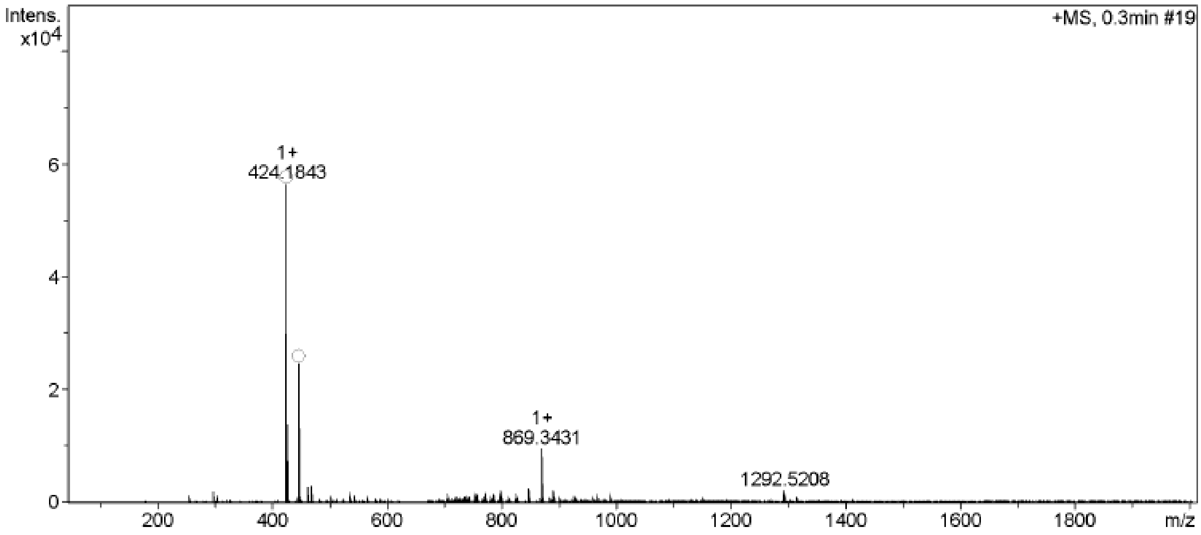

Meas. m/z	#	Ion Formula	m/z	err [mDa]	err [ppm]	rdb	N-Rule	e⁻ Conf	mSigma
424.184263	1	C19H22N9O3	424.184012	0.3	0.6	13.5	ok	even	3.7
446.165712	1	C19H21N9NaO3	446.165956	-0.2	-0.5	13.5	ok	even	3.3

SmartFormula Settings

Low value of mSigma indicates good isotopic pattern match

Spectrum 17 - HRMS ESI of 4-(((4-(2-aminopyrimidin-5-yl)-6-morpholino-1,3,5-triazin-2-yl)amino)methyl)-*N*-hydroxybenzamide (DRL-05) (**5e**).

Trinity College Dublin

School of Chemistry Mass Spectrometry Unit

Sample-ID		Station	
Submitter		Supervisor	
Analysis Name	DRL-006_GB2_01_43004.d	Acquisition Date	02/07/2025 17:26:21
Sample Description			

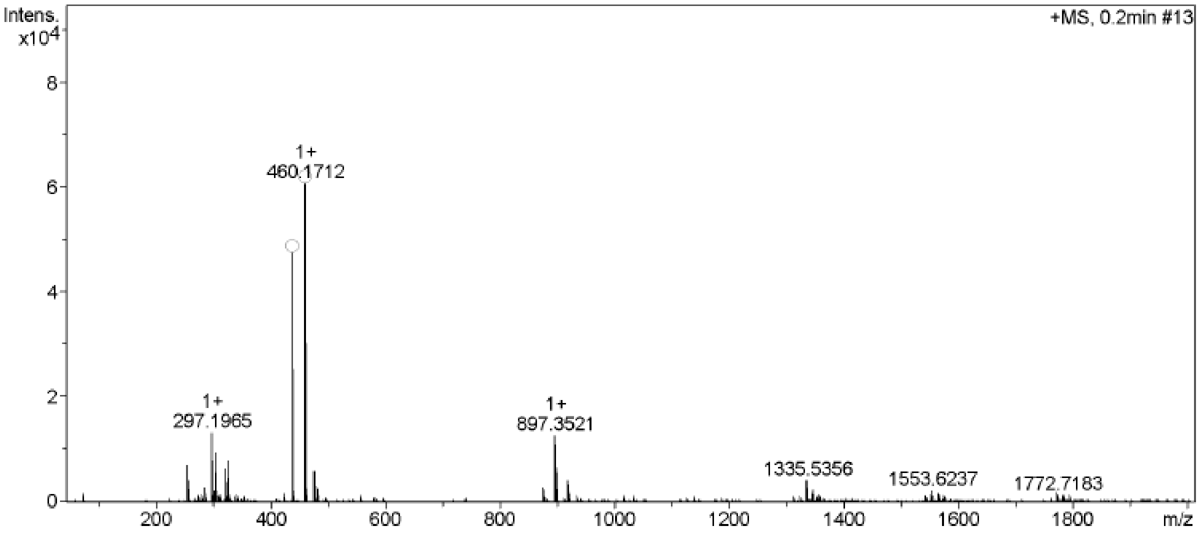

Meas. m/z	#	Ion Formula	m/z	err [mDa]	err [ppm]	rdb	N-Rule	e¯ Conf	mSigma
438.189448	1	C21H24N7O4	438.188429	1.0	2.3	13.5	ok	even	2.0
460.171157	1	C21H23N7NaO4	460.170373	0.8	1.7	13.5	ok	even	5.6

SmartFormula Settings

Low value of mSigma indicates good isotopic pattern match

Spectrum 18 - HRMS ESI of *N*-hydroxy-4-(((4-(6-methoxypyridin-3-yl)-6-morpholino-1,3,5-triazin-2-yl)amino)methyl)benzamide (DRL-06) (**5f**).

Purity by High-Performance Liquid Chromatography

Analysis Report

<Sample Information>

Sample Name : DRL-01
Sample ID : DRL-01
Data Filename : DRL-01.lcd
Method Filename : DAR_ACN50H2O50_15min.lcm
Batch Filename : DRL_purity.lcb
Vial # : 2
Sample Type : Unknown
Injection Volume : 10 uL
Date Acquired : 30/07/2025 10:25:08
Acquired by : System Administrator
Date Processed : 30/07/2025 10:59:11
Processed by : System Administrator

<Chromatogram>

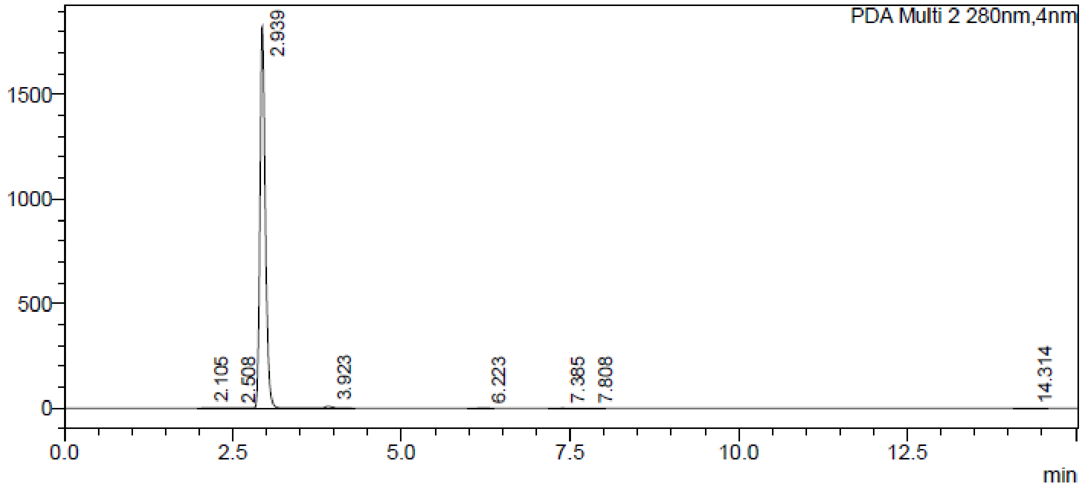

<Peak Table>

PDA Ch2 280nm

Peak#	Ret. Time	Area	Area%
1	2.105	16478	0.159
2	2.508	13507	0.131
3	2.939	10227689	98.981
4	3.923	64333	0.623
5	6.223	4126	0.040
6	7.385	2197	0.021
7	7.808	1743	0.017
8	14.314	2859	0.028
Total		10332932	100.000

Chromatogram 1 - Purity by HPLC for *N*-hydroxy-4-(((4-morpholino-6-phenyl-1,3,5-triazin-2-yl)amino)methyl)benzamide (DRL-01) (**5a**).

SHIMADZU LabSolutions

Analysis Report

<Sample Information>

Sample Name	: DRL-02		
Sample ID	: DRL-02		
Data Filename	: DRL-02.lcd		
Method Filename	: DAR_ACN50H2O50_15min.lcm		
Batch Filename	: DRL_purity.lcb		
Vial #	: 3	Sample Type	: Unknown
Injection Volume	: 10 uL		
Date Acquired	: 30/07/2025 10:43:04	Acquired by	: System Administrator
Date Processed	: 30/07/2025 11:00:35	Processed by	: System Administrator

<Chromatogram>

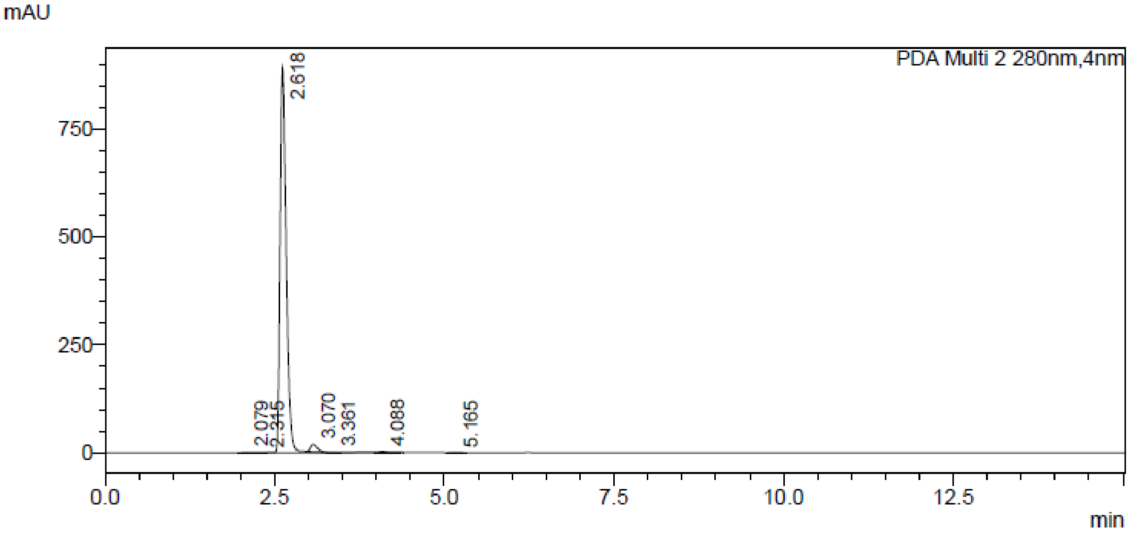

<Peak Table>

PDA Ch2 280nm

Peak#	Ret. Time	Area	Area%
1	2.079	7854	0.133
2	2.315	5624	0.095
3	2.618	5773627	97.460
4	3.070	115492	1.950
5	3.361	2365	0.040
6	4.088	17950	0.303
7	5.165	1174	0.020
Total		5924086	100.000

Chromatogram 2 - Purity by HPLC for *N*-hydroxy-4-(((4-(3-hydroxyphenyl)-6-morpholino-1,3,5-triazin-2-yl)amino)methyl)benzamide (DRL-02) (**5b**).

SHIMADZU LabSolutions

Analysis Report

<Sample Information>

Sample Name	: DRL-03		
Sample ID	: DRL-03		
Data Filename	: DRL-03.lcd		
Method Filename	: DAR_ACN50H2O50_15min.lcm		
Batch Filename	: DRL_purity.lcb		
Vial #	: 4	Sample Type	: Unknown
Injection Volume	: 10 uL		
Date Acquired	: 30/07/2025 11:01:04	Acquired by	: System Administrator
Date Processed	: 30/07/2025 11:21:15	Processed by	: System Administrator

<Chromatogram>

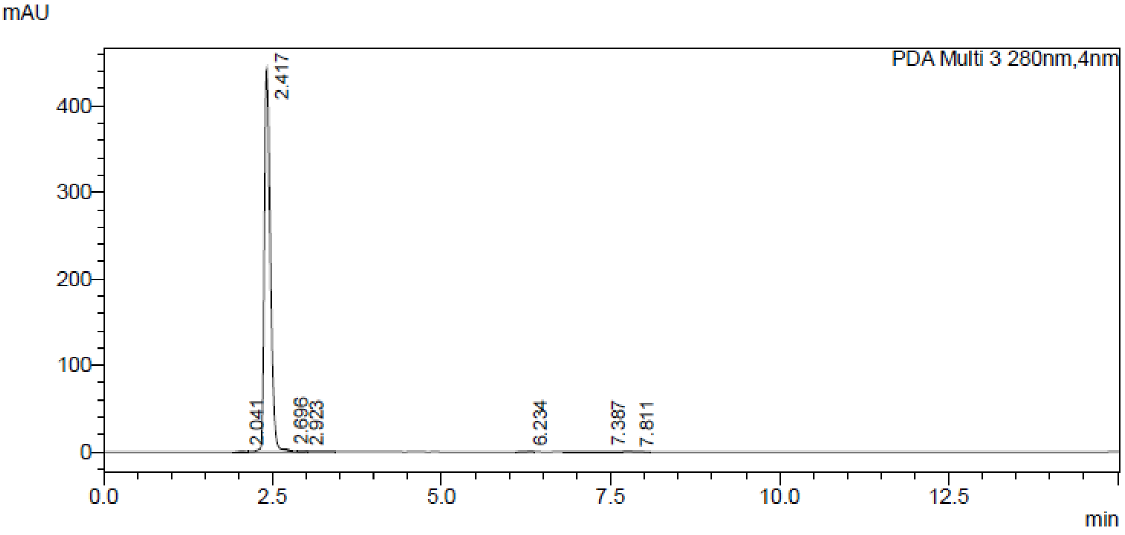

<Peak Table>

PDA Ch3 280nm

Peak#	Ret. Time	Area	Area%
1	2.041	6537	0.242
2	2.417	2670214	99.005
3	2.696	3165	0.117
4	2.923	1205	0.045
5	6.234	2360	0.087
6	7.387	11387	0.422
7	7.811	2175	0.081
Total		2697042	100.000

Chromatogram 3 - Purity by HPLC for *N*-hydroxy-4-(((4-morpholino-6-(pyridin-3-yl)-1,3,5-triazin-2-yl)amino)methyl)benzamide (DRL-03) (**5c**).

SHIMADZU LabSolutions

Analysis Report

<Sample Information>

Sample Name	: DRL-04		
Sample ID	: DRL-04		
Data Filename	: DRL-04.lcd		
Method Filename	: DAR_ACN50H2O50_15min.lcm		
Batch Filename	: DRL_purity.lcb		
Vial #	: 5	Sample Type	: Unknown
Injection Volume	: 10 uL		
Date Acquired	: 30/07/2025 11:19:01	Acquired by	: System Administrator
Date Processed	: 30/07/2025 12:14:29	Processed by	: System Administrator

<Chromatogram>

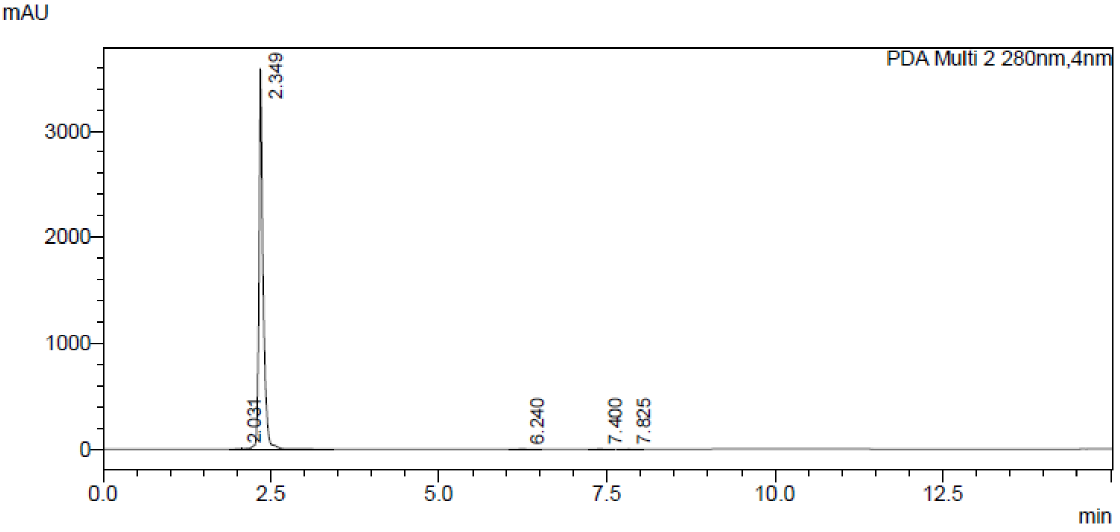

<Peak Table>

PDA Ch2 280nm

Peak#	Ret. Time	Area	Area%
1	2.031	18348	0.119
2	2.349	15351817	99.830
3	6.240	3972	0.026
4	7.400	2032	0.013
5	7.825	1739	0.011
Total		15377908	100.000

C:\LabSolutions\Data\DanielAlencar\2025\30072025\DRL-04.lcd

Chromatogram 4 - Purity by HPLC for 4-(((4-(6-aminopyridin-3-yl)-6-morpholino-1,3,5-triazin-2-yl)amino)methyl)-*N*-hydroxybenzamide (DRL-04) (**5d**).

SHIMADZU LabSolutions

Analysis Report

<Sample Information>

Sample Name	: DRL-05		
Sample ID	: DRL-05		
Data Filename	: DRL-05.lcd		
Method Filename	: DAR_ACN50H2O50_15min.lcm		
Batch Filename	: DRL_purity.lcb		
Vial #	: 6	Sample Type	: Unknown
Injection Volume	: 10 uL		
Date Acquired	: 30/07/2025 11:37:02	Acquired by	: System Administrator
Date Processed	: 30/07/2025 12:15:30	Processed by	: System Administrator

<Chromatogram>

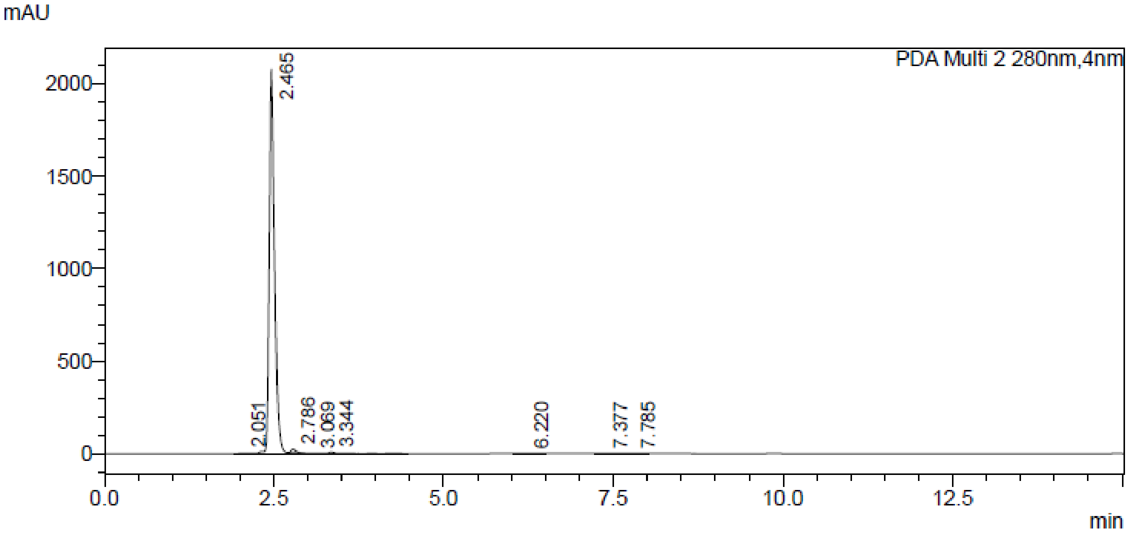

<Peak Table>

PDA Ch2 280nm

Peak#	Ret. Time	Area	Area%
1	2.051	20281	0.177
2	2.465	11285186	98.523
3	2.786	96869	0.846
4	3.069	1566	0.014
5	3.344	42866	0.374
6	6.220	4145	0.036
7	7.377	1949	0.017
8	7.785	1497	0.013
Total		11454360	100.000

Chromatogram 5 - Purity by HPLC for 4-(((4-(2-aminopyrimidin-5-yl)-6-morpholino-1,3,5-triazin-2-yl)amino)methyl)-*N*-hydroxybenzamide (DRL-05) (**5e**).

SHIMADZU LabSolutions

Analysis Report

<Sample Information>

Sample Name	: DRL-06		
Sample ID	: DRL-06		
Data Filename	: DRL-06.lcd		
Method Filename	: DAR_ACN50H2O50_15min.lcm		
Batch Filename	: DRL_purity.lcb		
Vial #	: 7	Sample Type	: Unknown
Injection Volume	: 10 uL		
Date Acquired	: 30/07/2025 11:54:59	Acquired by	: System Administrator
Date Processed	: 30/07/2025 12:16:24	Processed by	: System Administrator

<Chromatogram>

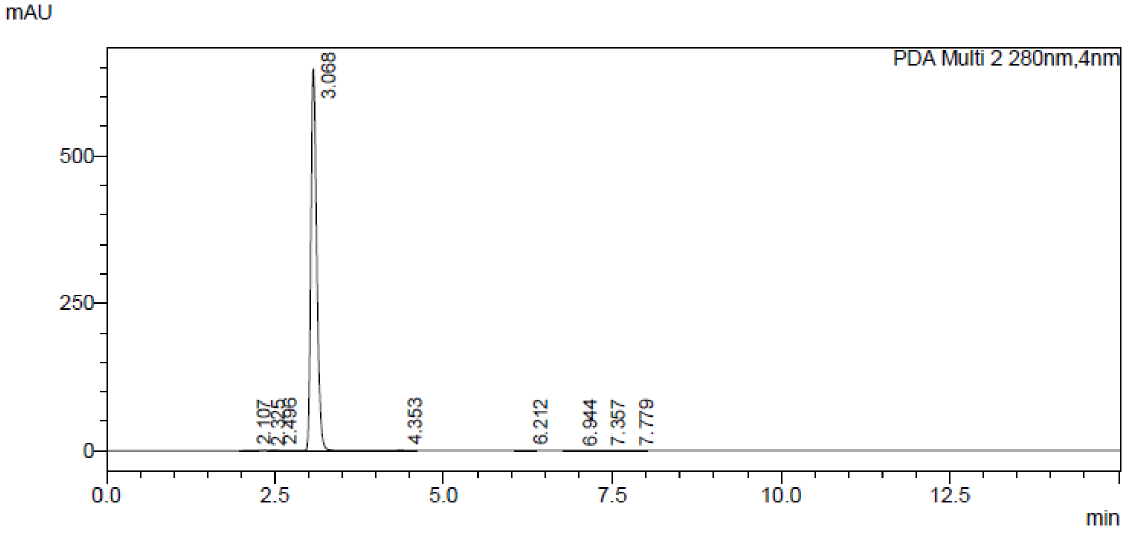

<Peak Table>

PDA Ch2 280nm

Peak#	Ret. Time	Area	Area%
1	2.107	5388	0.134
2	2.325	1866	0.046
3	2.496	12615	0.313
4	3.068	3985168	98.967
5	4.353	9638	0.239
6	6.212	2990	0.074
7	6.944	3891	0.097
8	7.357	3344	0.083
9	7.779	1878	0.047
Total		4026779	100.000

Spectrum 18 - Purity by HPLC for *N*-hydroxy-4-(((4-(6-methoxypyridin-3-yl)-6-morpholino-1,3,5-triazin-2-yl)amino)methyl)benzamide (DRL-06) (**5f**).
